# Cep295 is a conserved scaffold protein required for generation of a bona fide mother centriole

**DOI:** 10.1038/ncomms12567

**Published:** 2016-08-26

**Authors:** Yuki Tsuchiya, Satoko Yoshiba, Akshari Gupta, Koki Watanabe, Daiju Kitagawa

**Affiliations:** 1 Department of Molecular Genetics, Division of Centrosome Biology, National Institute of Genetics, Mishima, Shizuoka 411-8540, Japan; 2 Department of Genetics, School of Life Science, The Graduate University for Advanced Studies (SOKENDAI), Mishima, Shizuoka 411-8540, Japan

## Abstract

Centrioles surrounded by pericentriolar material (PCM) serve as the core structure of the centrosome. A newly formed daughter centriole grows into a functional mother centriole. However, the underlying mechanisms remain poorly understood. Here we show that Cep295, an evolutionarily conserved protein, is required for generation of a bona fide mother centriole organizing a functional centrosome. We find that Cep295 is recruited to the proximal centriole wall in the early stages of procentriole assembly. Cep295 then acts as a scaffold for the proper assembly of the daughter centriole. We also find that Cep295 binds directly to and recruits Cep192 onto the daughter centriole wall, which presumably endows the function of the new mother centriole for PCM assembly, microtubule-organizing centre activity and the ability for centriole formation. These findings led us to propose that Cep295 acts upstream of the conserved pathway for centriole formation and promotes the daughter-to-mother centriole conversion.

## Abstract (web summary)

Newly formed centrioles mature into functional mother centrioles. Here the authors show that the scaffolding protein Cep295 is recruited to the procentriole assembly site and assists daughter centriole assembly and daughter-to-mother centriole conversion.

## Graphical abstract

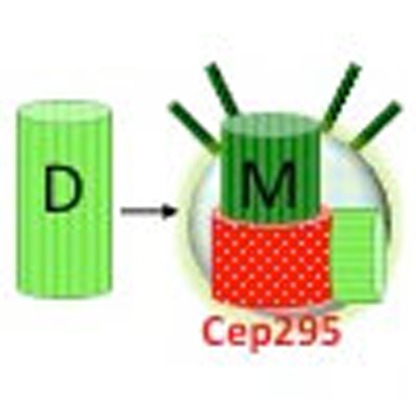

In most animal cells, centrosomes that consist of a pair of centrioles surrounded by amorphous pericentriolar material (PCM) act as the main microtubule-organizing centres (MTOCs). Formation of a daughter centriole near each mother centriole occurs once per cell cycle, which is required to maintain proper centrosome number. This process must be strictly regulated during cell cycle progression to ensure the robust formation of bipolar spindles and proper chromosome segregation during mitosis^1^,^2^,^3^. Indeed, aberration in centriole formation is implicated in human diseases such as cancer and ciliopathies^3^,^4^.

The daughter-to-mother centriole conversion is an essential event for generating a functional centrosome because, in this process, a daughter centriole recruits the PCM which is important for the microtubule nucleating activity of centrosomes. Moreover, only the mature mother centriole can generate a new centriole^5^. Previous studies have reported that the physical separation of the mother-daughter centriole pair, termed ‘disengagement', licenses centrioles to duplicate once per cell cycle^6^. However, the molecular mechanisms underlying daughter-to-mother centriole conversion after disengagement and how a mother centriole acquires the ability to form a new centriole in the next cell cycle are incompletely understood.

Concerning the evolutionarily conserved pathway for centriole formation, humans and *Caenorhabditis elegans* share five functional homologues, which are considered to be crucial factors for centriole formation: centrosomal protein of 192 kDa (Cep192)^7^,^8^, polo-like kinase 4 (Plk4)^9^,^10^, human spindle assembly abnormal-6 (HsSAS-6)^11^,^12^, SCL/TAL1 interrupting locus (STIL)^13^,^14^,^15^,^16^ and centrosomal P4.1-associated protein (CPAP)^17^,^18^,^19^ in humans. In the process of centriole formation in human cells, the presence of Cep192 and centrosomal protein of 152 kDa (Cep152)^20^,^21^,^22^ at centrioles is required for the centriolar recruitment of Plk4. At the onset of centriole formation, Plk4 phosphorylates STIL, which leads to the formation of a complex between the phosphorylated STIL and HsSAS-6 (refs 23, 24). This phosphorylation event promotes recruitment of the HsSAS-6-STIL complex to centrioles, which is followed by centriolar loading of CPAP for attachment of the centriolar microtubules and centriole elongation^17^,^18^,^19^. However, it is possible that other evolutionarily conserved factors critical for centriole formation have not yet been identified.

A previous study reported that centrosomal protein of 295 kDa (Cep295) coordinates only the centriole-to-centrosome conversion but does not affect centriole formation *per se* in human cells^25^. In addition, it has recently been shown that the Cep135-Cep295/Ana1-Cep152/Asl interactions enable the centriole-to-centrosome conversion in both *Drosophila melanogaster* and humans^26^. In this study, we identify Cep295 as a novel conserved factor acting upstream of Cep192 in centriole biogenesis. Cep295 appears to be recruited to the procentriole assembly site at the early stages of centriole duplication. Furthermore, we show that the interaction between Cep295 and Cep192 seems to be crucial for the integrity of centriole structure and also for daughter-to-mother centriole conversion.

## Results

### Cep295 is a conserved protein crucial for centriole assembly

**Figure 1:**
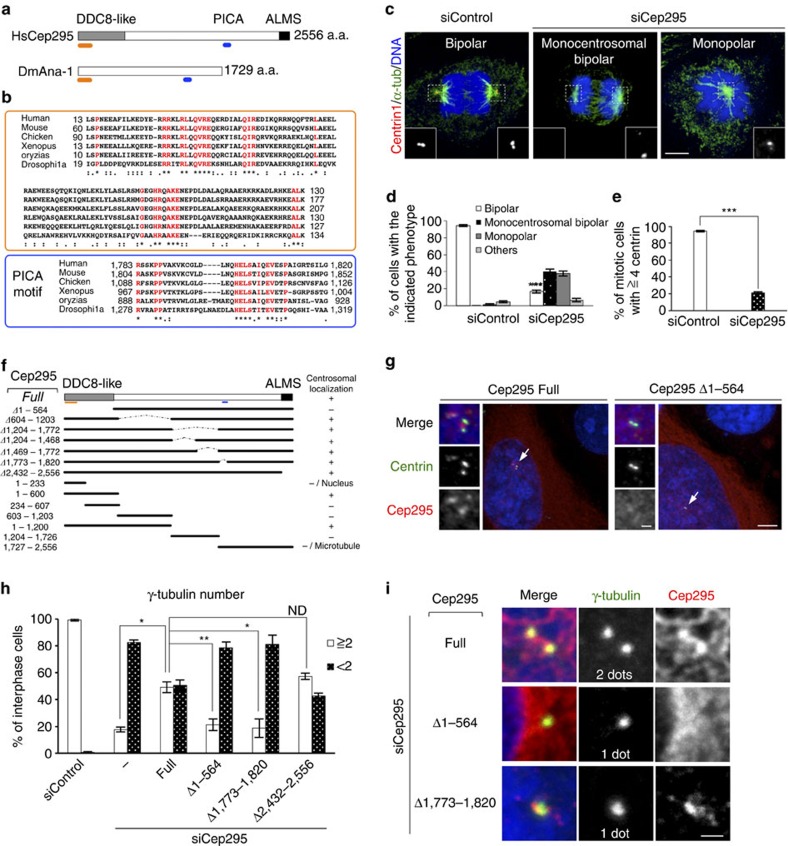
Cep295 was identified as an evolutionarily conserved protein required for centriole formation. (**a**) Schematic diagrams for Human Cep295 (HsCep295) and Drosophila Ana-1 (DmAna-1). The DDC8-like domain is shown in grey box, the ALMS domain in black box. The positions of two evolutionarily conserved domains are indicated in orange and blue lines, respectively. (**b**) Alignments of the evolutionarily conserved domains within human, mouse, chicken, Xenopus and Orizias Cep295 and Drosophila Ana1. The orange and blue boxes indicate the conserved region within the DDC8-like domain and PICA (Present in C-terminal of Ana-1) motif, respectively. Identical residues determined by Cluster W2 are shown in red. Asterisks indicate the residues identical in all aligned sequences; colons: conserved substitutions; periods: semi-conserved substitutions. (**c**–**e**) Cep295 ensures proper mitotic spindle and centriole formation. Cep295-depleted HeLa cells were stained with the indicated antibodies. Nuclei are shown in blue. Insets show approximately two-fold magnified views around the centrosome. Scale bar, 5 μm. Histograms represent frequency of mitotic cells with the indicated phenotype (**d**) or with ⩾4 centrin foci (**e**). Values are mean percentages±standard error of the mean (s.e.m) from three independent experiments (*N*=30 for each condition). ***P<0.001 (two-tailed *t*-test). (**f**,**g**) Schematic of full-length Cep295 and the deletion constructs used for immunofluorescence assay in HeLa cells in (**f**). All constructs encode siRNA-resistant forms of Cep295. The table shows presence (+) or absence (−) of centriolar localization of the deletion mutant proteins examined in the cells depleted of endogenous Cep295. In (**g**), DDC8-like domain (aa 1–564) is required for centriolar localization of Cep295. The cells were stained with the indicated antibodies. Nuclei are shown in blue. Arrows point to the centrioles. Scale bars, 5 μm in the low-magnified view, 1 μm in the inset. (**h**,**i**) For rescue experiments, Cep295-depleted HeLa cells were transfected with full-length Cep295 and the indicated mutants. The cells were stained with the indicated antibodies. Nuclei are shown in blue. Scale bar, 1 μm. Histograms represent frequency of interphase cells with the indicated number of γ-tubulin foci in each condition. Values are mean percentages±s.e.m from three independent experiments (*N*>50 for each condition). **P<0.01; *P<0.05; NS, not significant (two-tailed *t*-test).

Although it has been recently suggested that Cep295/KIAA1731 somehow regulates the centriole-to-centrosome conversion in human cells^25^, and also that sequential loading of Cep135, Cep295 and Cep152 onto daughter centrioles is needed for their maturation to become mother centrioles in *Drosophila* cells^26^, the exact function of Cep295 in centriole and centrosome biogenesis remains to be elucidated. Moreover, it is not clear whether its functional homologues in other species also play similar roles in these events. To determine whether Cep295 is a conserved factor involved in centriole formation across species, we first conducted a thorough BLAST analysis in eukaryotes. A previous study suggested that *Drosophila* Ana-1 (anastral spindle phenotype), which is implicated in centriole formation^27^,^28^,^29^ and human Cep295 appear to share a short homologous sequence^30^. Using an iterative BLAST search for the short stretch, we succeeded in identifying a 43-amino acid (aa) region of homology in other species (Fig. 1a,b). Accordingly, we termed the conserved short sequence as the ‘PICA (present in C-terminal of Ana-1)' motif. We also noted that Cep295 family proteins share another conserved region within the DDC8-like (differential display clone 8) domain at their N-terminus (Fig. 1a,b and Supplementary Fig. 1a).

Next, we found that RNAi-mediated depletion of Cep295 caused abnormal spindle formation and dramatically decreased the number of centrioles during mitosis in HeLa cells, compared to control mitotic cells that had four centrioles and assembled a bipolar spindle (21±1% of cells treated with siCep295, compared with 94±1% of control cells with ⩾4 centrioles; Fig. 1c–e). This result is in line with the previous observation that Cep295 is crucial for the maintenance of proper centriole numbers in human cells^25^. The similar defects in centriole number and mitotic spindle formation were also found when using RNAi targeting against different sequences of Cep295 ORF (Open Reading Frame) or in different human cell lines (Supplementary Fig. 1b). Note that the specificity of Cep295 siRNA was confirmed by using western blotting and immunofluorescence analysis, and also that Cep295 depletion did not affect cell cycle progression (Supplementary Fig. 1c,d,h).

We next aimed to identify which domains of Cep295 are required for its centriolar localization and for the assembly of centrosomes. We depleted endogenous Cep295 proteins using siRNA and expressed RNAi-resistant full-length or deletion constructs at comparable levels (Fig. 1f–i and Supplementary Fig. 1e–g,j). Whereas the number of centrosomes marked with γ-tubulin was reduced in most of the interphase cells upon depletion of endogenous Cep295, expression of full-length Cep295 functionally rescued this phenotype (17%±2% and 49%±4% of cells with ⩾2 centrosomes, respectively; Fig. 1f–i and Supplementary Fig. 1j). In contrast, a Cep295 mutant lacking the conserved DDC8-like domain did not localize to the centriole and failed to rescue the phenotype provoked by depletion of endogenous Cep295 (21%±4% of cells with ⩾2 centrosomes; Fig. 1f–i and Supplementary Fig. 1j). We found that another mutant lacking the PICA domain, but not the ALMS (Alstrom syndrome) motif, lost the ability to form centrosomes despite localizing to centrioles (17%±7% of cells with ⩾2 centrosomes; Fig. 1f,h,i and Supplementary Fig. 1j). We also noted that overly expressed Cep295 frequently colocalized with the microtubule networks through the C-terminal region including the ALMS domain (Supplementary Fig. 1i). These data suggest that the two conserved domains, DDC8-like and PICA, are critical for the function of Cep295 in the assembly of centrosomes and also that the centriolar localization of Cep295 is mediated by the DDC8-like domain.

### Cep295 localizes at the proximal end of centrioles

**Figure 2:**
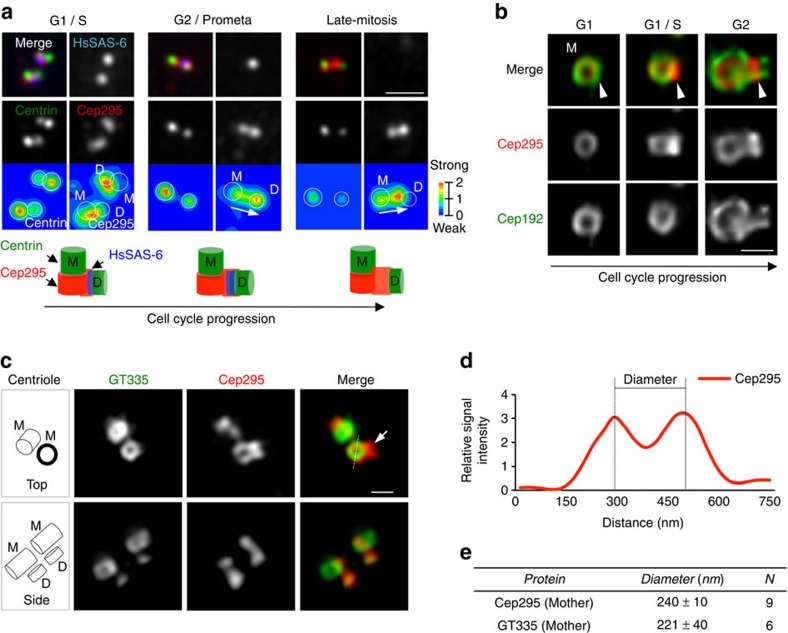
Subcellular localization of Cep295 throughout the cell cycle. (**a**) Centriolar distribution of Cep295 at different cell cycle stages. HeLa cells were stained with the indicated antibodies. The bottom panels represent quantification of the local signal intensity of centrin (left) and Cep295 (right). The local signal intensity was visualized in the indicated colours (M: mother centriole; D: daughter centriole). The schematic models represent the localization of Cep295, HsSAS-6 and centrin during the cell cycle. Note that HsSAS-6 disappears in late mitosis. In G1/S phase, the centrosomal linker connects the proximal end of two mother centrioles. Scale bar, 1 μm. (**b**) Cep295 localizes to the assembly site of procentrioles in the earliest stage (arrowheads) and forms a ring-like structure. The images were obtained by TCS SP8 HSR system using antibodies against Cep192 (green) and Cep295 (red). Scale bar, 500 nm. (**c**) 3D-SIM images representing top and side views of Cep295 at mother centrioles. GT335 was used as a centriole wall marker. An arrow points to recruitment of Cep295 onto the daughter centriole wall before the glutamylation of centriolar mitrotubules. Scale bar, 400 nm. (**d**,**e**) The graph shows the signal intensity of Cep295 at the mother centriole along the dotted line in (**c**). For quantification of the diameter, the distances between intensity maxima were measured (mean±s.d.).

To investigate the precise centriolar distribution of Cep295 across the cell cycle, we performed immunofluorescence analyses using specific antibodies against endogenous Cep295 (Supplementary Fig. 1d). Consistent with the recent study^25^, Cep295 gradually became enriched at daughter centrioles as the cell cycle progressed (Fig. 2a,b and Supplementary Fig. 2a). However, we also observed an intense signal of Cep295 on mother centrioles in G1 phase (Fig. 2b). To examine the detailed distribution of Cep295 on mother or daughter centrioles, we used three-dimensional structured illumination microscopy (3D-SIM). 3D-SIM analyses revealed that Cep295 localized at the proximal end of both daughter and mother centrioles and formed ring-like structures around the centriole wall (Fig. 2c). We also found that Cep295 was recruited to daughter centrioles before the appearance of a GT335 signal, which marks polyglutamylated centriolar microtubules. This observation suggests that the recruitment of Cep295 to daughter centrioles occurs in the early stages of procentriole formation. To measure the diameter of centriolar rings of Cep295 and GT335, we quantified the fluorescence intensity and determined the distance between the two peaks at opposite sides of the rings (240±10 nm; *n*=9 cells; Fig. 2d,e). Interestingly, the diameter of the Cep295 ring on mother centrioles was very close to that of GT335 (ref. 31) (221±40 nm; *n*=6 cells; Fig. 2e), which suggests that Cep295 is a component of the proximal part of the centriole wall. Furthermore, we found that the diameter of an N-terminal fragment of Cep295 fused with GFP at the C-terminus in mother centrioles is shorter than the diameter of endogenous Cep295 detected by a C-terminal-specific antibody (Supplementary Fig. 2b–d). This orientation of Cep295 at centrioles is consistent with the *Drosophila* Ana-1 orientation as previously described^26^, supporting the notion that the function of Cep295 for centriole formation is evolutionarily conserved.

### Cep295 is essential for maturation of new mother centrioles

**Figure 3:**
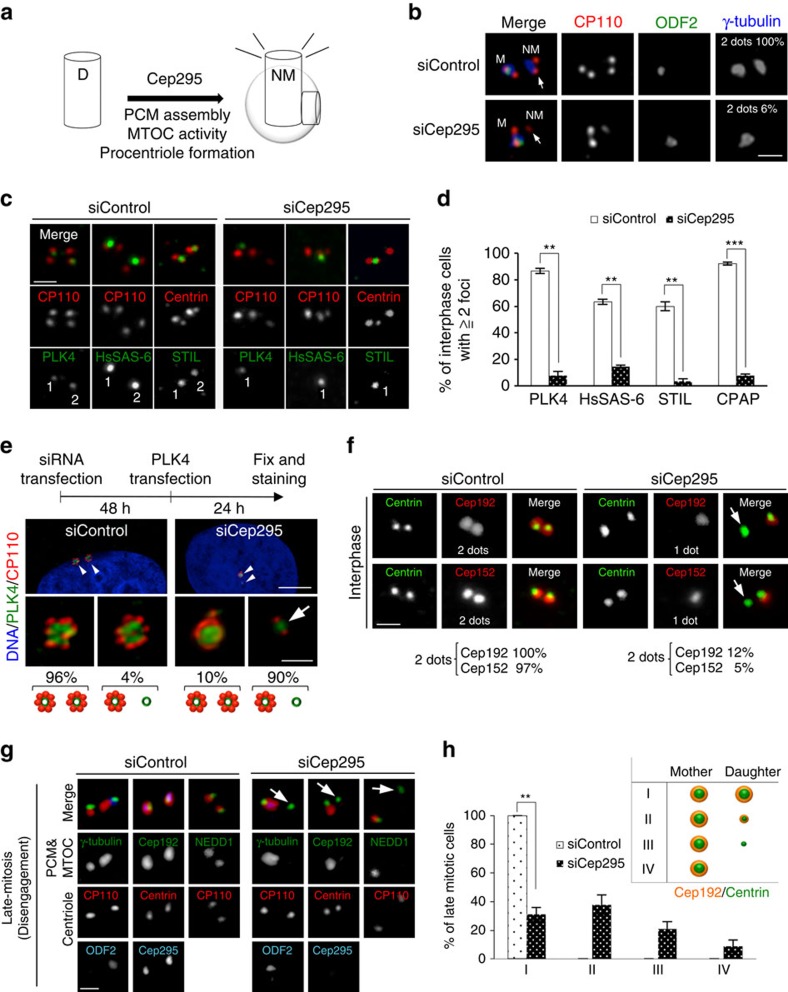
Cep295 is essential for the ability of a new mother centriole to recruit pericentriolar material and to generate a procentriole. (**a**) Schematic of Cep295 function described in this figure. (**b**) Three colour staining of centrioles in control and Cep295-depleted cells. The cells were stained with the indicated antibodies. ODF2 was used as an older mother centriole marker. Arrows point to a new mother centriole (M: older mother centriole; NM: new mother centriole). Scale bar, 1 μm. (**c**,**d**) HeLa cells transfected with control siRNA or Cep295 siRNA for 72 h were stained with indicated antibodies. CP110 and centrin were used as centriole markers^48^. Scale bar, 1 μm. (**c**) The number shown in the bottom panels represents the number of Plk4/HsSAS-6/STIL foci. (**d**) Histograms represent frequency of interphase cells with ≧2 Plk4/HsSAS-6/STIL/CPAP foci in each condition. Values are mean percentages±s.e.m from three independent experiments (*N*=30 for each condition). ***P<0.001; **P<0.01, (two-tailed *t*-test). (**e**) HeLa cells were treated with control siRNA or Cep295 siRNA, followed by transfection with an empty vector (−) or, pCMV5-Plk4ΔPEST-FLAG wild-type. The cells were stained with the indicated antibodies. An arrow points to the defect in multiple centriole formation induced by PLK4 over-expression, upon Cep295 depletion. Scale bars, 5 μm in the low-magnified view, 1 μm in the inset. Schematic illustrations show frequency of interphase cells with 2 or 1 over-duplicated centriole foci. (**f**) Cep295-depleted HeLa cells were stained with the indicated antibodies. Almost all control cells harbour ⩾2 Cep192 and ⩾2 Cep152 foci per cell, whereas only 12 and 5% of Cep295-depleted cells have ⩾2 Cep192 and ⩾2 Cep152 foci per cell, respectively. Scale bar, 1 μm. (**g**,**h**) To monitor the expression levels of Cep295 at the mother and daughter centrioles, the triple staining analysis was performed with the indicated antibodies as shown in the representative panels. HeLa cells were transfected with control or Cep295 siRNA for 24 h. (**g**) Arrows point to the defective recruitment of PCM components in the Cep295-depleted cells just after disengagement. Scale bar, 1 μm. (**h**) Histograms represent frequency of late mitotic cells with the indicated category in each condition. Values are mean percentages±s.e.m from three independent experiments (*N*=30 for each condition). **P<0.01 (two-tailed *t*-test).

The previous studies suggested that Cep295/Ana-1 depletion did not affect assembly of daughter centrioles *per se*, but perturbed their maturation to become new mother centrioles in the next cell cycle^25^,^26^. Although they showed that Cep295/Ana-1 is needed for the centriole-to-centrosome conversion in *Drosophila* and human cells, the mechanism by which Cep295 regulates this process still remains poorly understood. We therefore attempted to clarify the exact function of Cep295 in centrosome biogenesis (Fig. 3a). First, we confirmed that, in Cep295-depleted interphase cells, new mother centrioles were unable to recruit centrosomal proteins such as γ-tubulin. In contrast, older mother centrioles which were marked with Odf2 (ref. 32), an appendage marker of mature mother centrioles, could assemble functional centrosomes (Fig. 3b).

We also confirmed that procentriole formation at the new mother centriole was defective upon Cep295 depletion (Fig. 3b). We therefore sought to identify which step of the conserved pathway for procentriole formation is impaired at the new mother centriole in Cep295-depleted cells. Depletion of Cep295 affected centriolar loading of the critical factors, Plk4, STIL, HsSAS-6 and CPAP, to new mother centrioles in interphase cells, whereas that to older mother centrioles did not appear to be affected as much (Fig. 3c,d and Supplementary Fig. 3a,b). Conversely, depletion of Plk4, HsSAS-6 and STIL did not affect Cep295 localization to mother centrioles, even though procentriole formation was perturbed under these conditions, suggesting that Cep295 is recruited upstream of these factors (Supplementary Fig. 3c). To investigate further the different phenotypes at new and older mother centrioles upon Cep295 depletion, we tested the effects of Cep295 depletion on centriole overduplication induced by Plk4 overexpression in human cells. Consistently, we found that Cep295 depletion largely suppressed the formation of multiple procentrioles at new mother centrioles but not at older mother centrioles (Fig. 3e). Taken together, these data indicate that Cep295 is needed for the earlier stages of procentriole formation at new mother centrioles.

It is known that Cep192 coordinates the PCM assembly, which is essential for the organization of a functional centrosome with MTOC activity during mitosis. Cep192 is also a recruiter of Plk4 and Cep152, and is believed to be the most upstream of the evolutionarily conserved pathway for procentriole formation^33^,^34^. Interestingly, we found that depletion of Cep295 led to complete loss of Cep192 and Cep152 at only one of the two mother centrioles during interphase. This result suggests that, in the absence of Cep295, the new mother centriole lost the ability to hold essential proteins, Cep192 and Cep152, for PCM assembly and procentriole formation (Fig. 3f).

Next, to identify when in the cell cycle a new mother centriole becomes defective in centrosome assembly, we decided to examine the phenotype evoked by Cep295 depletion at an earlier time point (∼24 h after the RNAi treatment) and observe the redistribution of Cep192 to daughter centrioles just after mother-daughter centriole disengagement^6^ during the previous mitosis. By using triple staining analysis, we found that in control cells, Cep192 localized to both mother and daughter centrioles just after disengagement. Intriguingly, however, in the complete absence of Cep295 at both mother and daughter centrioles, we found that only mother centrioles marked with Odf2 harboured PCM proteins such as Cep192, NEDD1 and γ-tubulin whereas daughter centrioles did not recruit PCM proteins after centriole disengagement in late mitosis (Fig. 3g,h and Supplementary Fig. 3f). Consistent with this result, recruitment of other PCM proteins to daughter centrioles was already affected just after centriole disengagement in the absence of Cep295 (Fig. 3g and Supplementary Fig. 3d–f). While observing centriole disengagement in Cep295-depleted cells, we found that depletion of Cep295 caused precocious mother-daughter centriole disengagement during anaphase in mitosis, which may have occurred due to PCM loss at disengaged daughter centrioles (Supplementary Fig. 3g). Overall, these findings demonstrate that Cep295 is required for the transition of daughter centrioles to become functional mother centrioles from the earliest stages after centriole disengagement.

### Cep295 is crucial for the integrity of a daughter centriole

**Figure 4:**
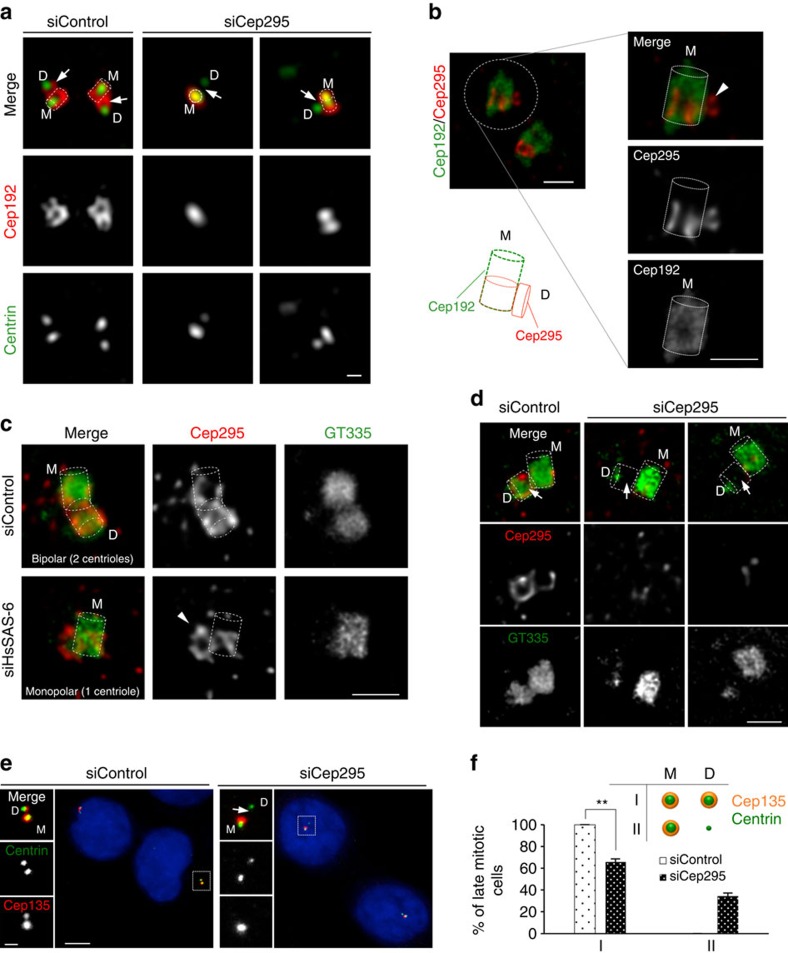
Cep295 is crucial for the integrity of a newly born daughter centriole at the older mother centriole. (**a**) 3D-SIM images showing centriolar distribution of Cep192 in control and Cep295-depleted HeLa cells (M: mother centriole; D: daughter centriole). Arrows point to the daughter centriole wall (*N*=5). Dotted lines indicate the shape of centriole cylinders in this figure. Scale bar, 400 nm. (**b**) U2OS cells were transfected with Flag-tagged Cep192 plasmid for 24 h. The cells were fixed and stained with antibodies against Flag and Cep295 for STED microscope. The right panels show magnified views. Arrowhead points to the cap-like structure of Cep295 at the procentriole assembly site before recruitment of Cep192 (*N*=10). Scale bar, 500 nm. The schematic illustrates the centriolar localization of Cep295 and Cep192. (**c**) STED images showing centriolar distribution of Cep295 in the presence or absence of HsSAS-6. HeLa cells were treated with control siRNA or HsSAS-6 siRNA for 48–60 h and stained with the indicated antibodies. To make sure the efficacy of HsSAS-6 depletion, we chose the Hs-SAS-6-depleted cells having only one mother centriole with a mono-polar spindle. Arrowhead shows centriolar distribution of Cep295 without the cartwheel structure (*N*=3). Scale bar, 500 nm. (**d**) Control and Cep295-depleted HeLa cells were visualized by STED microscope using the indicated antibodies. Arrows point to poly-glutamylation of centriolar microtubules stained with GT335 antibody at the daughter centrioles (*N*=3). Scale bar, 500 nm. (**e**,**f**) (**e**) HeLa cells treated with control siRNA or siRNA targeting endogenous Cep295 for 24 h were stained with antibodies against centrin1 (green) and Cep135 (red; a proximal component of centrioles). Nuclei are shown in blue. The arrow indicates defective recruitment of Cep135 to the disengaged daughter centriole. Scale bars, 5 μm in the low-magnified view, 1 μm in the inset. (**f**) Histograms represent frequency of late-mitotic cells with the indicated category in each condition. Values are mean percentages±s.e.m. from three independent experiments (*N*=30 for each condition). **P<0.01, (two-tailed *t*-test).

Why was the daughter centriole already unable to recruit PCM components after centriole disengagement in Cep295-depleted cells? A previous study claimed that Cep295 is crucial for the centriole-to-centrosome conversion^25^, but not for centriole formation itself. However, this conclusion seemed to be drawn from experiments that used a limited number of centriole markers against newly formed centrioles in Cep295-depleted cells. Given that upon Cep295 depletion, Cep192 was not present at the daughter centrioles that had just disengaged from mother centrioles (Fig. 3g,h), we assumed that formation of the daughter centrioles might be already defective. These considerations prompted us to reexamine whether Cep295 depletion leads to defects in the formation of daughter centrioles. In control cells, 3D-SIM analyses revealed that Cep192 was recruited to the wall of newly formed daughter centrioles that were engaged with mother centrioles. In stark contrast, we found that the recruitment of Cep192 to the daughter centriole wall did not occur in Cep295-depleted cells (Fig. 4a). We also found that the arrangement of Cep192 at the mother centriole was affected in the same condition even though some Cep192 still remained at the mother centriole. Although the upstream factors regulating centriolar targeting of Cep192 remain unknown, these data indicate that Cep295 is crucial for the initial recruitment of Cep192 at a newly born daughter centriole.

Next, to precisely monitor the order in which Cep295 and Cep192 are recruited to the assembly site of procentrioles, we used the gated Stimulated Emission Depletion (STED) microscopy. This technique enabled us to distinguish the distribution of the two proteins at procentrioles from that at the mother centrioles (Fig. 4b). We analysed centriolar localization of the two proteins in human G1 cells, and found that Cep295 was loaded to the procentriole assembly site even while Cep192 localization was limited at the mother centrioles. This observation implies that Cep295 could provide a platform for assembly of the proximal part of a procentriole. To address this idea, we next examined whether this centriolar recruitment of Cep295 occurs in the absence of the cartwheel structure, which serves as the base of the procentriole. Upon depletion of HsSAS-6, an essential component of the cartwheel structure^35^,^36^,^37^,^38^, a portion of Cep295 appeared to be recruited onto the mother centriole wall and formed a cap-like structure, presumably at the potential assembly site of a procentriole (Fig. 4c). This result suggests that centriolar recruitment of Cep295 occurs independently of the cartwheel formation. Taken together, these data support the notion that Cep295 provides a platform for the proximal part of procentrioles in the early stages of centriole formation, and acts upstream of the evolutionarily conserved pathway for centriole formation.

Given the above results, we postulated that Cep295 is implicated in the assembly of the proximal part of the centriole structure. The gated STED microscopy analyses demonstrated that newly formed daughter centrioles exhibited a significant reduction in the number of polyglutamylated centriolar microtubules, as marked by GT335, especially at the proximal part (Fig. 4d). We then tested the integrity of the cartwheel structure upon Cep295 depletion. To monitor this, we quantified the fluorescence signal intensity of HsSAS-6 at centrioles. Although centriolar recruitment of HsSAS-6 was detectable in Cep295-depleted interphase cells, the signal intensity of HsSAS-6 was considerably weaker in these Cep295-depleted interphase cells than in control cells (Supplementary Fig. 4a–c). We next looked at Cep135, a proximal centriolar component^39^ and intriguingly found that depletion of Cep295 led to complete loss of Cep135 from the daughter centrioles just after centriole disengagement (Fig. 4e,f). This is in line with the previous finding that depletion of Ana-1 also affected Cep135 recruitment to daughter centrioles in *Drosophila* cells^26^. On the other hand, we could detect only 34% reduction of Cep295 at centrioles by Cep135 depletion in human cells (Supplementary Fig. 4f–j). Overall, we conclude that Cep295 is critical for proper formation of daughter centrioles. Given the previous study^25^ and our observation (Supplementary Fig. 4d,e) that Cep295 regulates the stability of a new mother centriole, we speculate that incomplete daughter centriole formation by Cep295 depletion could lead to instability and defective function of the resulting new mother centriole in the next cell cycle.

### The Cep295-Cep192 interaction promotes centrosome biogenesis

**Figure 5:**
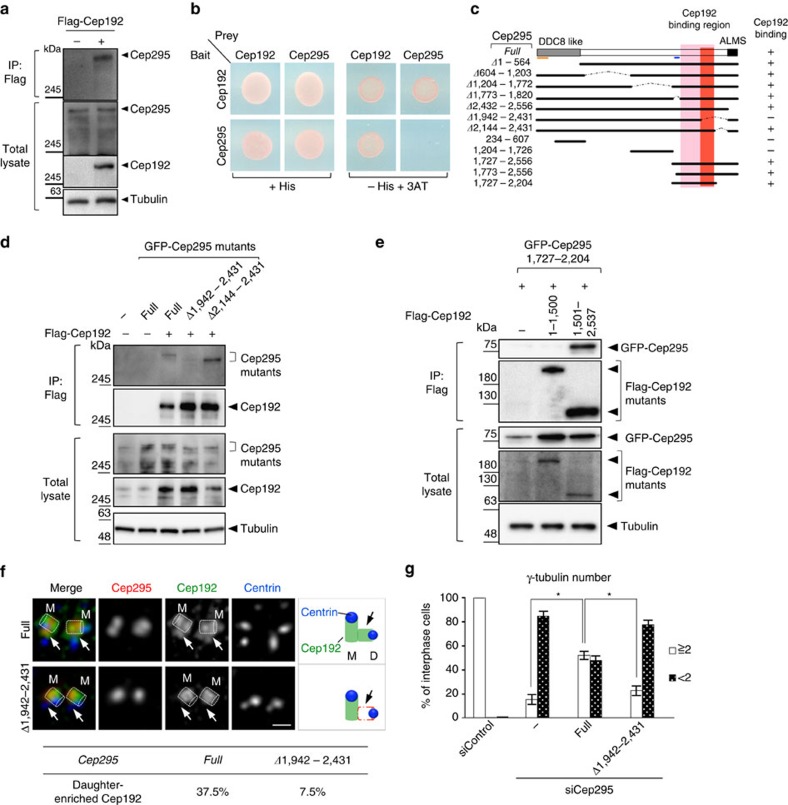
Interaction between Cep295 and Cep192 is required for the recruitment of Cep192 to a newly formed daughter centriole. (**a**) Physical interaction between endogenous Cep295 and Cep192. U2OS cells were transfected with Flag-tagged Cep192 full-length for 24 h. The proteins were then immunoprecipitated from cell lysates with Flag beads. Total cell lysate and immunoprecipitates (IPs) were analysed by western blotting using Flag, Cep295 or α-tubulin (loading control) antibodies. The same result was obtained in 293T cells. (**b**) Yeast two-hybrid assay showing the interaction between full-length Cep192 and full-length Cep295. The indicated clones were grown on the plates lacking histidine and containing 50 mM 3-AT at 30 °C. The same results were obtained from two independent clones for each combination. The outcome of the other clone is not shown. (**c**) Schematic of full-length Cep295 and the deletion mutants used for co-IP assays with Flag-tagged full-length Cep192 in U2OS cells. The sufficient and necessary regions for Cep192-binging in Cep295 are represented in pink and red, respectively. The DDC8-like domain, ALMS domain, and two evolutionarily conserved domains are indicated. (**d**,**e**) Co-immunoprecipitation assay in U2OS cells testing interaction between Flag-Cep192 and the indicated Cep295-GFP deletion mutants or between Cep295 fragment containing the Cep192-interacting region and N-terminal or C-terminal fragment of Cep192. The Flag-tagged proteins were immunoprecipitated using Flag beads from the cell lysate. Total cell lysates and IPs were analysed by western blotting using the indicated antibodies. (**f**) Three colour staining of Cep295-depleted HeLa cells expressing full-length Cep295 and the mutant protein lacking aa 1942–2431. The phenotype was analysed mostly in the first round of cell cycle (∼48 h after the RNAi treatment). The cells were analysed by TCS SP8 HSR system using antibodies against Cep192 (green), Cep295 (red) and CP110 (blue). Arrows point to the daughter centriole wall. M: mother centriole indicated as a cylinder. Scale bar, 1 μm. (**g**) For rescue experiments, Cep295-depleted HeLa cells were transfected with full-length Cep295 and the indicated mutant. Histograms represent frequency of interphase cells with the indicated number of γ-tubulin foci in each condition. Values are mean percentages±s.e.m from three independent experiments (*N*>50 for each condition). *P<0.05 (two-tailed *t*-test).

To understand how Cep295 recruits Cep192 to a newly formed daughter centriole, we tested whether Cep295 interacts with Cep192. Remarkably, immunoprecipitation (IP) assays in human cells and yeast two-hybrid analyses revealed this to indeed be the case (Fig. 5a,b). We noted that the expression of Cep295 increased the protein levels of Cep192 in a concentration-dependent manner *in vivo*, which suggests that Cep295 may also stabilize Cep192 (Supplementary Fig. 5a). Using a co-IP assay and yeast two-hybrid analysis with deletion constructs of Cep295, we narrowed down the Cep295 region required for Cep192-binding to the short stretch spanning aa 1942–2144 (Fig. 5c,d and Supplementary Fig. 5b–d).

We also found that Cep295 fragments containing the binding region are sufficient for binding to the C-terminal region of Cep192 in human cells (Fig. 5e and Supplementary Fig. 5c,d). We therefore reasoned that the interaction between the two proteins might be needed for recruitment of Cep192 onto the daughter centriole wall. We depleted endogenous Cep295 using siRNAs and expressed full-length Cep295 or deletion constructs lacking the Cep192-interacting region at comparable levels in HeLa cells. As expected, we found that expression of the Cep295 mutant that is unable to bind Cep192 could not recruit Cep192 onto the wall of a newly formed daughter centriole (Fig. 5f). To address whether these defective daughter centrioles could become functional mother centrioles in the next cell cycle, we focused on the recruitment of γ-tubulin to the resulting new mother centriole. While depletion of endogenous Cep295 caused a significant reduction of γ-tubulin foci in most interphase cells, the expression of full-length Cep295 could functionally rescue this phenotype (15%±4% and 52%±3% of cells with ⩾2 centrosomes, respectively; Fig. 5g). In contrast, a Cep295 mutant lacking the Cep192-interacting region failed to rescue the phenotype provoked by depletion of endogenous Cep295 (22%±4% of cells with ⩾2 centrosomes; Fig. 5g). Taken together, these data strongly suggest that the interaction between Cep295 and Cep192 is required for daughter-to-mother centriole conversion.

**Figure 6:**
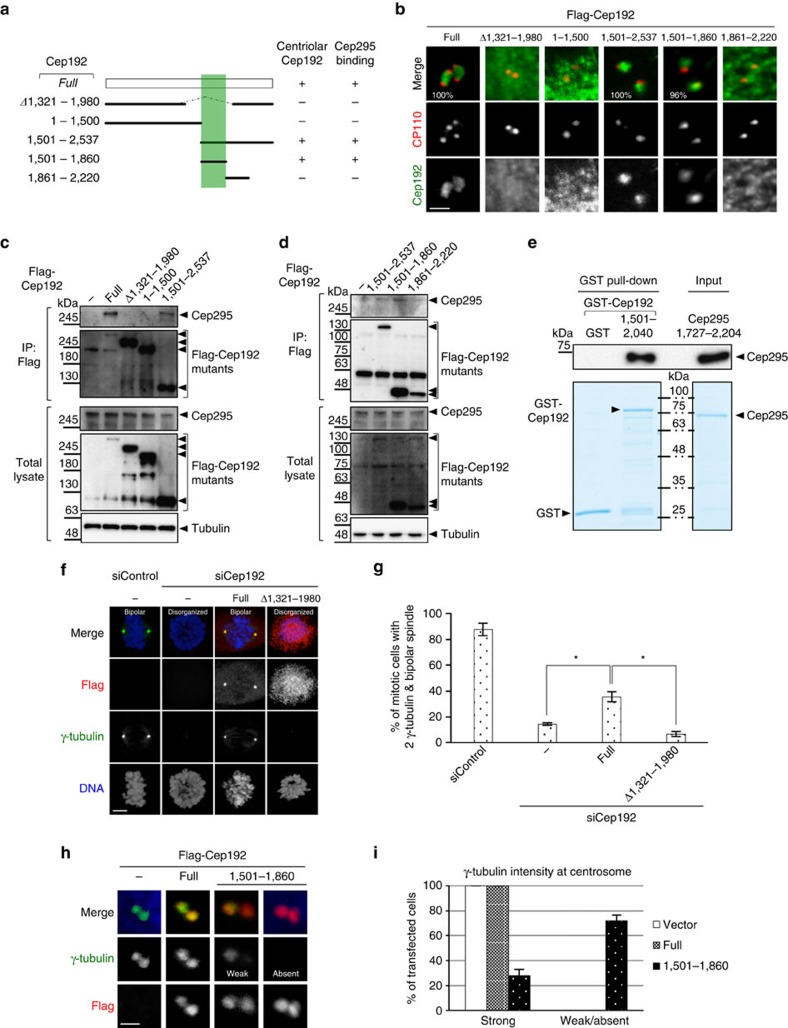
Interaction between Cep295 and Cep192 is required for centriolar localization of Cep192. (**a**) Schematic of full-length Cep192 and the deletion mutants used for co-IP assays with endogenous Cep295 in U2OS cells. The minimal Cep295-binging region in Cep192 is represented in grey. (**b**) U2OS cells were transfected with Flag-Cep192 full-length and the indicated mutants. The cells were stained with antibodies against Cep192 (green) and CP110 (red). Scale bar, 1 μm. (**c**,**d**) U2OS cells expressing Flag-Cep192 full length or the deletion mutant proteins were immunoprecipitated with FLAG beads. Total cell lysates and IPs were analysed by western blotting using Cep295, Flag or tubulin antibodies. (**e**) GST pull-down assay showing the interaction between Cep295 and Cep192 fragments (aa 1727–2204 of Cep295 and aa 1501–2040 of Cep192) *in vitro.* These bacterially purified recombinant proteins contain the interacting regions that were identified by co-immunoprecipitation experiments. (**f**,**g**) For rescue experiments, Cep192-depleted U2OS cells were transfected with RNAi-resistant full-length Cep295 and the indicated mutant. Scale bar, 5 μm. Histograms represent frequency of bipolar mitotic spindles with the indicated number of γ-tubulin foci. Values are mean percentages±s.e.m from three independent experiments (*N*=30 for each condition). *P<0.05 (two-tailed *t*-test). (**h**,**i**) Dominant negative effects of the Cep192 fragment that binds to Cep295. U2OS cells were transfected with full-length Cep295 and the indicated mutant. Scale bar, 1 μm. Histograms represent frequency of transfected cells with the indicated intensity of γ-tubulin foci. Values are mean percentages±s.e.m from three independent experiments (*N*=30 for each condition). **P<0.01 (two-tailed *t*-test).

We next sought to identify the region of Cep192 for binding to Cep295 and for its centriolar targeting. Firstly, by using immunofluorescence analyses with Cep192 deletion mutants, we found that a short fragment of Cep192 (aa 1501–1860) was sufficient for its loading to the centriole in human cells. In contrast, the Cep192 mutants that lack regions containing aa 1501–1860 did not localize to the centriole (Fig. 6a,b and Supplementary Fig. 6a,b). Secondly, consistent with these observations, co-IP analyses revealed that the Cep192 mutants lacking the region of aa 1501–1860 failed to interact with endogenous Cep295 (Fig. 6a,c). In contrast, the C-terminal fragments of Cep192 containing the region of aa 1501–1860 were capable of binding to endogenous Cep295 (Fig. 6a,d). Thirdly, to confirm the Cep295-Cep192 interaction *in vitro* with the bacterially purified recombinant proteins, we purified Cep295 and Cep192 fragments (aa 1727–2204 of Cep295 and aa 1501–2040 of Cep192) containing the interacting regions that were identified by co-IP experiments. We demonstrated that the two proteins interact with each other by GST pull-down assays (Fig. 6e), suggesting the physical association between Cep295 and Cep192. Next, to address whether the centriolar targeting of Cep192 is required for centriole-to-centrosome conversion, we knocked down endogenous Cep192 and expressed a Cep192 deletion mutant that fails to associate with Cep295. We found that the Cep192 mutant did not rescue recruitment of γ-tubulin at centrosomes and mitotic spindle formation in the cells depleted of endogenous Cep192 (Fig. 6f,g) whereas the full-length of Cep192 rescued the phenotypes. This result supports the notion that the Cep295-Cep192 interaction is needed for centriolar recruitment of Cep192 and centriole-to-centrosome conversion. Furthermore, we conducted an additional experiment using the short fragment of Cep192 that binds to Cep295. Interestingly, expression of the Cep192 fragment that localized to centrioles inhibited centrosomal recruitment of PCM components such as γ-tubulin and endogenous Cep192 (Fig. 6h,i and Supplementary Fig. 6c,d). This result implies the dominant negative effect of the Cep192 fragment that masked the Cep192-interaction site of centriolar Cep295, on centriolar recruitment of endogenous Cep192 and on PCM assembly. Overall, these findings strongly suggest that Cep192 is recruited to the newly formed daughter centriole by binding to Cep295, and that this interaction is necessary for centriole-to-centrosome conversion.

## Discussion

**Figure 7:**
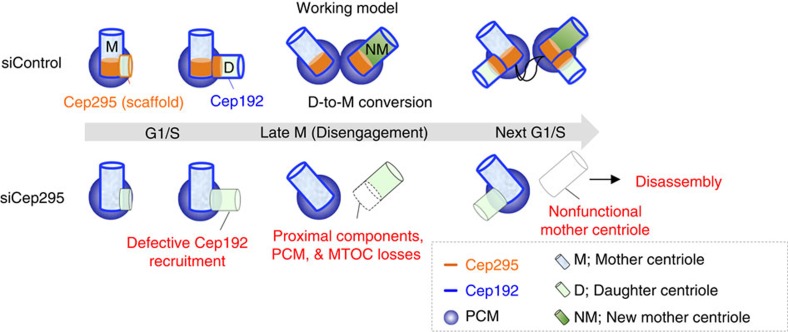
Cep295 ensures daughter-to-mother centriole conversion via Cep192 recruitment onto the newly formed daughter centriole wall. A speculative model of the role of Cep295 in centriole biogenesis. Cep295 promotes recruitment of Cep192 onto the wall of a newly formed daughter centriole. Cep295 is also critical for the integrity of the proximal part of the daughter centriole and for stability of the resulting centriole in the next cell cycle. The events coordinated by Cep295 are thus critical for the ability of a new mother centriole to duplicate, recruit PCM components and act as the MTOC.

In conclusion, our work indicates that Cep295 promotes the recruitment of Cep192 onto daughter centrioles, which is a critical step for daughter-to-mother centriole conversion (Fig. 7). In addition, Cep295 depletion results in defects in the structure of the proximal parts of daughter centrioles. Consistent with the previous study, we also confirmed that such compromised daughter centrioles finally disassembled during interphase in the next cell cycle.

How can Cep295 localize to daughter centrioles? Our study suggests that Cep295 is present at the potential assembly site of a procentriole even before cartwheel formation (Fig. 4). Similarly in *Chlamydomonas*, the amorphous ring structure is generated before the appearance of the cartwheel^40^,^41^. Even though we could not find a potential homolog of Cep295 in *Chlamydomonas*, it is tempting to speculate that Cep295 provides a similar function serving as a platform to promote the initiation of procentriole assembly. It will be interesting to investigate whether Cep295 molecules on the mother centriole are rearranged and moved to the assembly site of a procentriole to trigger centriole formation. It should also be noted that while this study was under review, another studies reported that Cep295 and Ana1 regulate centriole elongation in human^42^ and *Drosophila* cells^43^, respectively.

Although it has recently been claimed that the interaction between Cep135-Ana1/Cep295-Asterless/Cep152 is required for centriole-to-centrosome conversion^26^, this interaction could not sufficiently explain the function of Cep295 in recruiting PCM components at centrioles in humans. This molecular network seems to be critical for centriole formation in *Drosophila* cells. However, given that both Cep152 and Cep135 are not known to be essential for PCM assembly in human cells^20^,^21^,^22^,^44^, it is unlikely that the Cep135/Cep295/Cep152 complex is responsible for the conversion of daughter centrioles into the functional mother centrioles that organize PCM in human cells. Moreover, this interaction cannot explain the reduction of Cep192 at centrioles upon Cep295 depletion, since Cep192 is known to be upstream of centriolar loading of Cep152 (refs 33, 34) in human cells. In contrast, as shown in our study, the defects in centriolar loading of Cep192 following Cep295 depletion can lead to the reduction of Cep152 at centrioles. These considerations lead us to propose that the Cep295-Cep192 interaction is fundamental for the functions of Cep295 in centriole-to-centrosome conversion, because this interaction could potentially enable the mother centrioles to duplicate, recruit PCM and nucleate microtubules.

How could older mother centrioles duplicate without Cep295? We speculate that although Cep295 is essential for the initial recruitment of Cep192 to the daughter centriole through direct interaction between the two proteins, another mechanism including perhaps the self-assembly of Cep192 may contribute to the maintenance of Cep192 at centrioles. Indeed, some Cep192 proteins still remained at the older mother centriole even in the absence of Cep295 (Fig. 3). It has recently been shown that Cep192 recruits Plk4 for centriole formation and assembles PCM around mother centrioles^33^,^34^. We therefore speculate that, in the absence of Cep295, the remnant Cep192 is sufficient to recruit critical centriole components, such as Plk4, STIL and HsSAS-6, at the procentriole assembly site on the older mother centriole to promote centriole formation even though the integrity of the resulting daughter centriole may be affected. In addition, we also noticed that overexpression of Cep295 did not induce centriole overduplication, suggesting that Cep295 is important for ensuring the proper formation of a new daughter centriole, but not sufficient for generating extra daughter centrioles. Based on the findings in this study, we propose that Cep295 acts upstream of the conserved pathway for centriole formation and promotes the daughter-to-mother centriole conversion. Further studies will be needed to explain how the physical interaction between Cep295 and Cep192 helps to initiate the assembly of a functional centrosome.

## Methods

### Cell culture and transfection

HeLa, U2OS and HEK293T cells were obtained from the ECACC (European collection of cell cultures). To generate HeLa cells stably expressing GFP-centrin1 (ref. 45), centrin1 cDNA was subcloned into the pEGFP-N1 vector (Clontech). GFP-centrin1 fusion proteins were expressed and then isolated by using the limited dilution method with 500 μg ml^−1^ G418. The cell lines used in this study have been authenticated by STR profiling in ECACC. All cells were cultured in Dulbecco's modified Eagle's medium) containing 10% fetal bovine serum at 37 °C in a 5% CO_2_ atmosphere.

Transfection of siRNA or DNA constructs into HeLa, U2OS and HEK293T cells was conducted using Lipofectamine RNAiMAX (Life Technologies) or Lipofectamine 2000 (Life Technologies), respectively. Unless otherwise noted, the transfected cells were analysed 48–72 h after transfection with siRNA and 24 h after transfection with DNA constructs.

### RNA interference

The following siRNAs were used: Silencer Select siRNA (Life Technologies) against Cep295 #1 (s229742), Cep295 #2 (s229743), Cep192 #1 (s226819), Cep192 #2 (s30227), STIL (s12863), SAS-6 (s46487), CPAP (s31623), Cep135 (s18587) and negative control #1 (4390843); custom siRNA (Sigma Genosys) against 3′UTR of Plk4 (5′- CTCCTTTCAGACATATAAG -3′); custom siRNA (Nihon Bio Co., Ltd.) against Cep152 (ref. 46) and Cep135 (refs 26, 44). Unless otherwise noted, Cep295 #1, Cep192 #1 and custom siRNA (Nihon Bio Co., Ltd.) against Cep135 (refs 26, 44) were used in this study.

### Plasmids

The full-length Cep295 was amplified from cDNA library of HeLa cells. Note that the Cep295 clone lacks the exon 11 of the full-length sequence registered in the NCBI. The Cep295 cDNA was subcloned into the pCMV5 vectors for expression of N-terminal Flag-tagged, C-terminal GFP-tagged or non-tagged proteins, respectively, in human cells. The pCMV5-Cep295 deletion mutant constructs, such as Δ1–564, Δ604–1203, Δ1204–1772, Δ1204–1468, Δ1469–1772, Δ1773–1820 and Δ2432–2556, were created using PrimeSTAR mutagenesis basal kit (TaKaRa) according to manufacturer's protocol. The pCMV5-Cep295-GFP deletion mutant constructs, such as Δ1942–2431 and Δ2144–2431, were created using PrimeSTAR mutagenesis basal kit (TaKaRa). The Cep295 fragments, such as 1–233, 1–600, 234–607, 603–1203, 1204–1726, 1727–2556 and 1773–2556, were subcloned into the pCMV5-Flag vector, and 1–233, 1–600, 1–1200 and 1727–2204 were subcloned into the pCMV5-GFP vector.

The pcDNA3-Flag construct encoding full-length Cep192 was kindly provided by Erich A Nigg. The constructs for expression of Flag-tagged Cep192 deletion mutant proteins were generated with PrimeSTAR mutagenesis basal kit (TaKaRa). The pcDNA3 construct encoding full-length PLK4-Flag was kindly gifted from Hiroyuki Mano.

### Antibodies

The following primary antibodies were used in this study: Rabbit polyclonal antibodies against Cep295/KIAA1731 (Sigma, HPA038596, IF 1:1000, WB 1:1000), Cep192 (a gift from Laurence Pelletier, IF 1:1000), Cep192 (Bethyl laboratories, A302–324A, WB 1:1000), Cep152 (Bethyl laboratories, A302–480A, IF 1:1000), Cep152 (Bethyl laboratories, A302–479A, WB 1:1000), CP110 (a gift from Brian David Dynlacht, IF 1:500), CP110 (proteintech, 12780–1-AP, IF 1:500), CPAP (a gift from Pierre Gönczy, IF 1:500), CPAP/CENP-J (Proteintech, 11517-1-AP, IF 1:500), STIL (Abcam, ab89314, IF 1:500, WB 1:1000), Cep135 (Abcam, ab196809, IF 1:1000); mouse monoclonal antibodies against centrin-2 (Millipore, 20H5, IF 1:1000), HsSAS-6 (Santa Cruz Bio-technology, Inc., sc-81431, IF 1:500, WB 1:1000), Plk4 (Merck Millipore, clone 6H5, MABC544, IF 1:500), γ-tubulin (GTU88) (Sigma-Aldrich, T5192, IF 1:1000), Polyglutamylation Modification (GT335, mAb) (AdipoGen, AG-20B-0020-C100, IF 1:5000), FLAG-tag (Sigma, F1804, IF 1:1000, WB 1:1000) and α-tubulin (Sigma-Aldrich, DM1A, IF1:1000); goat polyclonal antibody against GFP, FITC-conjugated (Abcam, ab6662, IF 1:300). Alexa 488- labelled Cep192 (Bethyl laboratories, A302–324A, IF 1:200) and ODF2 (Abcam, ab43840, IF 1:200) were generated with Alexa Fluor labelling kits (Life Technologies) and used for three colour staining in Figs. 3b and 5f. The following secondary antibodies were used: Alexa Fluor 488 goat anti-mouse IgG (H+L) (Molecular probes, A-11001, 1:500), Alexa Fluor 568 goat anti-rabbit IgG (H+L) (Molecular probes, A-11011, 1:500) for IF; Alexa Fluor 555 goat anti-rabbit IgG (H+L) (Molecular probes, A-21428, 1:500) for STED; Goat polyclonal antibodies-horseradish peroxidase against mouse IgG (Promega, W402B, 1:5 000), rabbit IgG (Promega, W401B, 1:5000) for WB. For detection of exogenous Cep295, because there was a technical difficulty to detect the N-terminal tag of full-length Cep295, we used Cep295 antibody.

### Microscopy

For immunofluorescence analysis, the cells cultured on coverslips (Matsunami: No 1 for confocal microscope, No 1 s for SIM, STED microscope) were fixed using −20 °C methanol for 7 min and washed with PBS. The cells were permeabilized after fixation with PBS/0.05% TritonX-100 (PBSX) for 5 min three times, and incubated for blocking in 1% BSA in PBSX for 30 min at room temperature (RT). The cells were then incubated with primary antibodies for 24 h at 4 °C, washed with PBSX three times, and incubated with secondary antibodies for 1 h at RT. The cells were thereafter washed with PBSX twice, stained with 0.2 μg ml^−1^ Hoechst 33258 (DOJINDO) in PBS for 5 min at RT, washed again with PBSX and mounted onto glass slides.

Counting the number of immunofluorescence signals was done using an Axioplan2 fluorescence microscope (Carl Zeiss) with a 100 × /1.4 NA plan-APOCHROMAT objective. Quantification of the signal intensity in Fig. 2a and supplementary Fig. 4a was performed using DeltaVision Personal DV-SoftWoRx system (Applied Precision) equipped with a CoolSNAP CH350 CCD camera. Serial section images along the z-axis were stacked with the ‘quick projection' algorithm in softWoRx. The signal intensities of centriolar Cep295, Centrin, and HsSAS-6 were quantified with the Data Inspector tool in softWoRx. We assessed cells from several fields for each experiment. The investigators were normally blinded to the sample ID during experiments and outcome assessment. Once a field was determined, we counted all cells which match the criteria within the field. In the experiments using the cells expressing Flag-tagged full-length or mutants of Cep295, we counted cells adequately expressing the Cep295 proteins at comparable levels and excluded cells expressing the Cep295 proteins at low levels or cells excessively-expressing the Cep295 proteins.

Confocal microscopy images were taken by the Leica TCS SP8 HSR system equipped with a Leica HCX PL APO × 63/1.4 oil CS2 objectives and excitation wavelength 405, 488 and 561 nm. To obtain high-resolution images, the pinhole was adjusted at 0.5 airy units. Scan speed was set to 200 Hz in combination with five-fold line average in 856 × 100 format (pixel size 43 nm). The images were collected at 130 nm z steps. For deconvolution, Huygens essential software (SVI; Scientific Volume Imaging) was used. In Fig. 3e,f, values are mean percentages from three independent experiments (*N*=30 for each condition). In Fig. 5f, values are mean percentages from two independent experiments (*N*=20 for each condition).

STED images were taken by a Leica TCS SP8 STED 3X system with a Leica HC PL APO 100 × /1.40 oil STED WHITE, and 660 nm gated STED. Scan speed was set to 100 Hz in combination with five-fold line average in a 512 × 80 format (pixel size 15–20 nm). The images were collected at 180 nm z steps. The STED images were processed by deconvolution with Huygens professional software (SVI). The resolution of green signal (∼80 nm, Alexa488) is generally lower than that of red signal (∼50 nm, Alexa555) in this system. In this study, we mainly looked at our protein of interest at centrioles in red whereas the other protein was visualized in green.

3D-SIM images were taken by the Nikon N-SIM imaging system with Piezo stage, Apo TIRF 100 × oil objective lens (NA1.49), excitation wavelength 488 and 561 nm, and iXon DU-897 EMCCD camera (Andor Technology Ltd.), The images were collected at 100 nm z steps. Quantification of the signal intensity and reconstruction in Figs. 2c–e, 4a were performed using NIS-Elements AR software.

### Immunoprecipitation and western blotting

For preparation of human cell lysates for western blotting, HEK293T and U2OS cells were collected 24 h after transfection, washed in PBS and lysed on ice for 20 min in lysis buffer (20 mM Tris/HCl pH7.5, 50 mM NaCl, 1% TritonX-100, 5 mM EGTA, 1 mM DTT, 2 mM MgCl_2_ and 1/1000 protease inhibitor cocktail (nakarai tesque)). Insoluble material was removed after centrifugation for 15 min at 15,000 r.p.m. For IP of Flag-tagged Cep192 proteins, whole cell lysates were incubated with Flag antibody-conjugated M2 agarose (SIGMA) for 2 h or over night at 4 °C. The beads were washed at least three times with lysis buffer and resuspended in SDS-sample buffer before loading onto 5–12% polyacrylamide gels, followed by transfer on Immobilon-P membrane (Millipore corporation). The membrane was probed with the primary antibodies, followed by incubation with their respective horseradish peroxidase-conjugated secondary antibodies (Promega). Washes were performed in PBS containing 0.02% Tween (PBST). The signal was detected with Chemi Doc XRS+ (BIO RAD). Signal intensity of immuno-reactive bands was measured using Adobe Photoshop. Unless otherwise specified, the experiments of western blotting were repeated at least three times. The antibody against α-tubulin was used as a loading control. Uncropped scans for all western blots were represented in Supplementary Fig. 7.

### Yeast two-hybrid analysis

Full-length or fragments of Cep192 or Cep295 were cloned into the modified version of the vectors pSM671 (bait) and pSM378 (prey) (gifts from Satoru Mimura). Yeast strain L40 was grown in complete medium (yeast extract peptone dextrose; (YPD)) and transformed with the indicated vectors. Positive colonies were grown on plates lacking leucine and tryptophan in the presence of histidine at 30 °C. After a few days, cells were streaked on plates containing 50 mM 3-amino-1,2,4-triazole (Sigma) without leucine, tryptophan and histidine. The plates were incubated at 30 °C for a few days. The same results were obtained from two independent clones for each combination. Yeast two-hybrid analysis was repeated at least three times.

### *In vitro* binding assay

Cep295 or Cep192 fragments were cloned in pGEX system vectors (GE healthcare) encoding for GST-tags. This analysis was performed as described previously^47^ with slight modifications. The recombinant protein expression of the fragments was performed in *E. coli* strain BL21 gold (DE3) in LB medium. Protein expression was induced at 18 °C by addition of 0.3 mM IPTG and allowed to proceed for 18 h. Cell pellets expressing GST-Cep295 and Cep192 fragments (aa 1727–2204 of Cep295 and aa 1501–2040 of Cep295) were lysed by lysozyme treatment and sonication, resuspended in lysis buffer containing 50 mM Tris-HCl (pH 7.5), 500 mM NaCl, 5 mM EDTA, 1 mM DTT, 1:1000 protease inhibitor cocktail (Sigma) and 0.5% TritonX-100. The lysates were incubated with Glutathion sepharose beads (GE healthcare). The beads were then washed ten times with lysis buffer supplemented with additional 500 mM NaCl at high salt concentrations. Cep295 fragment proteins were then eluted from the beads by removal of the GST-tags by prescission protease (GE healthcare) in a buffer containing 20 mM Tris-HCl (pH 7.5), 150 mM NaCl, 0.5 mM EDTA, 1 mM DTT.

For GST pull-down assay, the Glutathion sepharose beads which retain bacterially purified GST-Cep192 fragment were resuspended in ice-cold lysis buffer and incubated with ∼5 μg bacterially purified Cep295 for 2 h at 4 °C. The resin was washed seven times with ice-cold lysis buffer and resuspended in SDS sample buffer. Proteins were separated by SDS-PAGE and stained with SimplyBlue Safe (Invitrogen).

### Live cell imaging

A Confocal Scanner Box, Cell Voyager CV1000 (Yokogawa Electric Corp) equipped with a 63 × oil immersion objective lens and the stage incubator for 35 mm dish was used for live cell imaging. HeLa cells stably expressing GFP-centrin1 were treated with control siRNA or Cep295 siRNA for 24 h and cultured on 35 mm glass-bottom dishes (MatTek Co.) at 37 °C in a 5% CO_2_ atmosphere. Images were taken by Back-illuminated EMCCD camera. After 24 h from transfection, the cells were visualized every 10 min over 24–48 h. To count the number of centrin foci, the images were collected at 1 μm z steps (from 25 to 30 Z-planes and generated using ImageJ (National Institutes of Health)).

### Data availability

The data that support the findings of this study are available from the corresponding author upon request.

## Additional information

**How to cite this article:** Tsuchiya Y. *et al*. Cep295 is a conserved scaffold protein required for generation of a bona fide mother centriole. *Nat. Commun.* 7:12567 doi: 10.1038/ncomms12567 (2016).

## Supplementary Material

Supplementary Information Supplementary Figures 1-7
