# Supplementary material for: Cep295 is a conserved scaffold protein required for generation of a bona fide mother centriole
Source: Nat Commun. 2016 Aug 26;7:12567. doi: 10.1038/ncomms12567 (PMC5007451; doi:10.1038/ncomms12567)
Supplement: Supplementary Information — Supplementary Figures 1-7 [file ncomms12567-s1.pdf]

Supplementary Figure 1

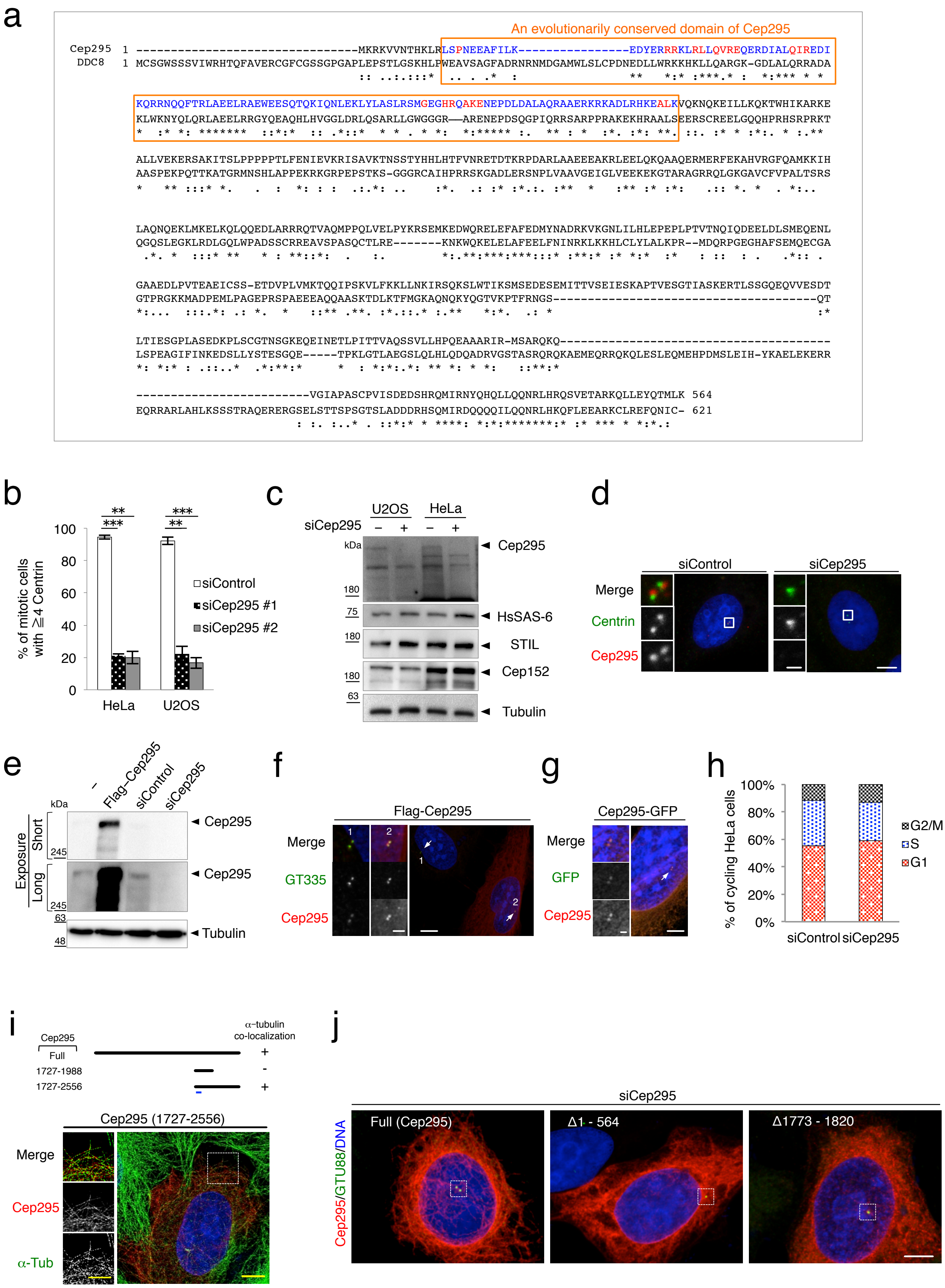

Supplementary Figure 1. Cep295 is a conserved protein for centriole formation.

(a) Alignments of DDC8 and the N-terminal domain of Cep295, performed with ClustalW2. The orange box indicates the PINA (Present in N-terminal of Ana-1) motif. Evolutionarily conserved residues are colored in red and others in blue. Asterisks indicate the residues identical in the aligned sequences; colons: conserved substitutions; periods: semi-conserved substitutions. **(b)** Cep295 is required for maintenance of the proper centriole number. HeLa and U2OS cells were treated with control siRNA or siRNA targeting against different sequences of Cep295 for 72 hours. Histograms represent frequency of mitotic cells with ≥4 centrin foci. Values are mean percentages ± s.e.m from three independent experiments (*N* = 30 for each condition). ***, $P < 0.001$; **, $P < 0.01$ (two-tailed *t*-test). **(c,d)** The efficiency of siRNA targeting Cep295 and the specificity of Cep295 antibody were confirmed by WB and immunofluorescence analyses. (c) HeLa and U2OS cells were treated with control siRNA or siRNA targeting Cep295 for 48 hours. Total cell lysates were analyzed by western blotting using Cep295, HsSAS-6, STIL, Cep152, or α-Tubulin antibodies, as indicated. The same result was obtained in 293T cells. (d) Cep295-depleted HeLa cells were stained with the indicated antibodies. Nuclei are shown in blue. Insets show approximately three-fold magnified views around the centrosome. Scale bars, 5 μm in the low-magnified view, 1 μm in the inset. **(e-g)** The exogenous expression of full-length Cep295 in human cells was assessed by WB and immunofluorescence analyses. (e) U2OS cells were transfected with an empty vector (–) or pCMV5 vector encoding Flag-tagged Cep295 full-length, and treated with control siRNA or siRNA targeting endogenous Cep295 for 48 hours. Total cell lysates were analyzed by western blotting using Cep295 or α-Tubulin antibodies. (f,g) Immunofluorescence analysis revealed that Cep295 full-length proteins tagged with Flag at N-terminus (in (f)) or with GFP at C-terminus (in (g)) localized to the centriole. U2OS cells expressing the tagged Cep295 proteins were stained with the indicated antibodies. Nuclei are shown in blue. Insets show the magnified views of centrioles (arrows). Scale bars, 10 μm in the low-magnified view, 2 μm in the inset. 1: weakly expressed Cep295, 2: highly expressed Cep295 (exogenous). (f). Scale bars, 5 μm in the low-magnified view, 1 μm in the inset (g). **(h)** No significant difference in cell cycle between control and Cep295-depleted HeLa cells. The cell cycle phase of each control or Cep295-depleted cell was judged by the pattern of PCNA, centrin and DAPI staining. G1: interphase cells without PCNA; S: interphase cells with PCNA; G2: interphase cells with separate pairs of centrin foci; mitosis: mitotic cells with separate pairs of centrin foci and condensed nuclei. **(i)** Expression of C-terminal region of Cep295 in human cells leads to formation of filamentous structures. U2OS cells were transfected with pCMV5 vector encoding Cep295 full-length, aa 1727-1988 or aa 1727-2556 for 24 hours. The cells were fixed and stained with the indicated antibodies. Note that Cep295 fragment (aa 1727-2556) co-localized with α-Tubulin. Insets show approximately two-fold magnified views. Scale bars, 5 μm in the low-magnified view, 2 μm in the inset. **(j)** The low-magnified images shown in Fig. 1i. Scale bar, 5 μm.

Supplementary Figure 2

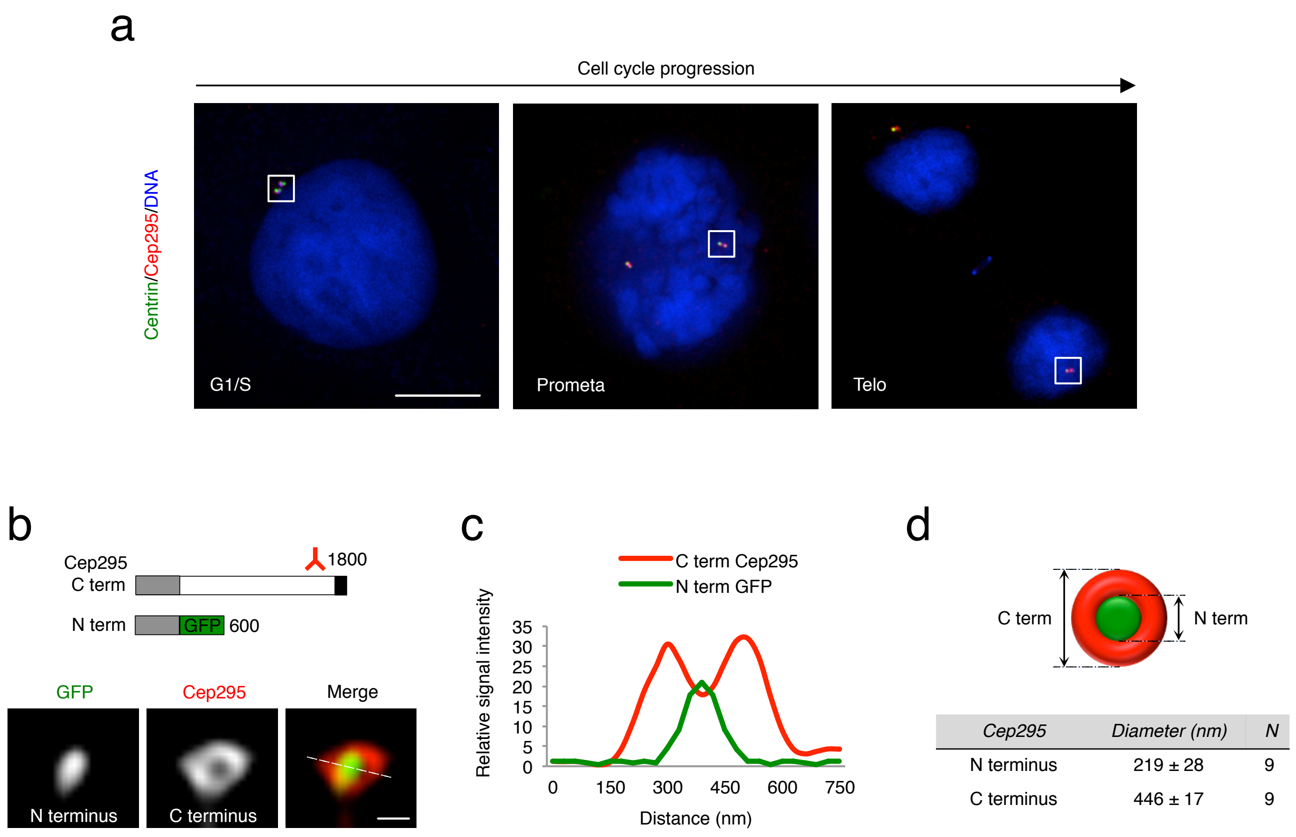

Cep295	*Diameter (nm)*	*N*
N terminus	219 ± 28	9
C terminus	446 ± 17	9

Supplementary Figure 2. Centriolar distribution of Cep295 across the cell cycle.

(a) HeLa cells at different stages of the cell cycle were stained with the indicated antibodies. Nuclei are shown in blue. Magnification of the insets is shown in Fig. 2a. Scale bar, 5 μm. **(b)** Schematic diagrams for Cep295. The DDC8-like domain is shown in gray box, the ALMS domain in black box. To investigate the orientation of Cep295 within the centriole structure, we marked the N-terminal region of Cep295 with GFP-tag and the C-terminal region with a specific antibody raised against aa 1831-1932 of Cep295. Since tagging at the N-terminus of Cep295 somehow affects its localization and expression, the indicated Cep295 short fragment tagged with GFP at its C-terminus was used for marking the Cep295 N domain.

The images representing top view of Cep295 at mother centrioles were obtained by TCS SP8 HSR system using antibodies against GFP (green) and Cep295 (red). Scale bar, 200 nm. **(c)** The graph shows the signal intensity of Cep295 at the mother centriole along the dotted line in (b). For quantification of the diameter, the distances between intensity maxima were measured. **(d)** Schematic representation of a top view of Cep295 at the mother centriole. The external diameter was measured for quantification (mean ± s.d.).

Supplementary Figure 3

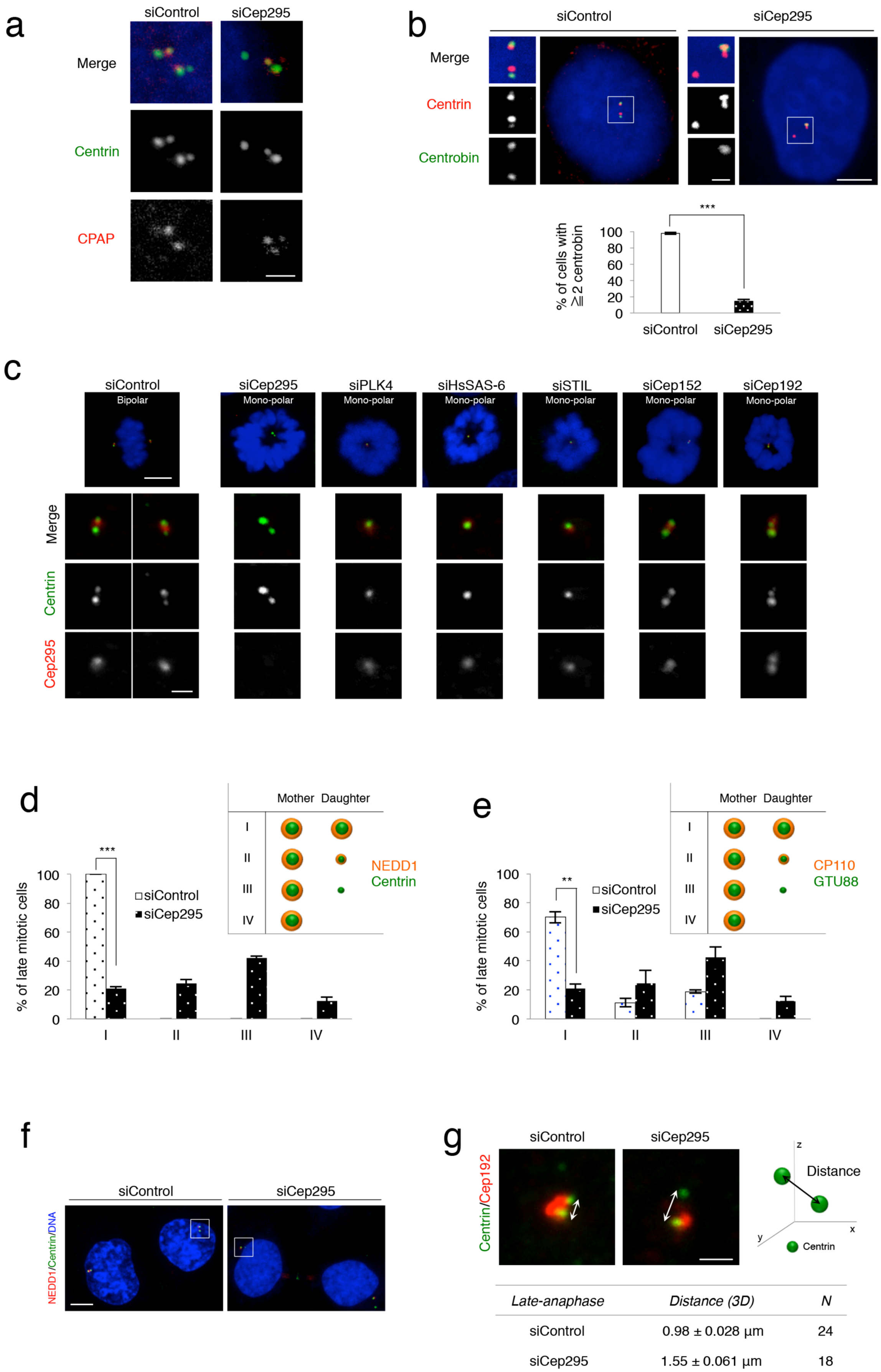

Late-anaphase	*Distance (3D)*	*N*
siControl	0.98 ± 0.028 µm	24
siCep295	1.55 ± 0.061 µm	18

Supplementary Figure 3. Cep295 is crucial for a new mother centriole to generate a procentriole and organize a functional PCM.

(a,b) CPAP and centrobin were reduced at the new mother centriole in Cep295-depleted cells. HeLa cells transfected with control siRNA or Cep295 siRNA for 72 hours were stained with the indicated antibodies. Nuclei are shown in blue. Histograms represent frequency of interphase cells with ≥2 centrobin foci. Values are mean percentages ± s.e.m from three independent experiments (N = 30 for each condition). ***, $P < 0.001$, (two-tailed t-test). Scale bar, 1 μm (a). Scale bars, 5 μm in the low-magnified view, 1 μm in the inset (b). **(c)** Depletion of critical factors for centriole formation did not affect Cep295 localization at the mother centriole. HeLa cells transfected with control siRNA or the indicated siRNAs for 48 hours were stained with antibodies against centrin1 (green) or Cep295 (red). Nuclei were shown in blue. To make sure the efficiency of each RNAi treatment, we chose the mitotic cells having only one or two centrin foci and thus being indicative of defects in centriole formation. Scale bars, 5 μm in the low-magnified view, 1 μm in the inset. **(d,e)** Quantification of the experiments shown in Fig. 3g. Histograms represent frequency of late mitotic cells with the indicated category. Values are mean percentages ± s.e.m from three independent experiments (N = 30 for each condition). ***, $P <$ 0.001; **, $P < 0.01$, (two-tailed t-test). **(f)** Late mitotic HeLa cells transfected with control siRNA or Cep295 siRNA, were stained with the indicated antibodies. Magnification of the insets is shown in Fig. 3g. Scale bar, 5 μm. **(g)** Depletion of Cep295 causes early disengagement during late mitosis. HeLa cells treated with control or Cep295 siRNA for 24 hours were stained with antibodies against centrin1 (green) and Cep192 (red). The 3D distances between the two centrin foci were measured for late-anaphase centrioles (mean ± s.d.). Scale bar, 1 μm.

Supplementary Figure 4

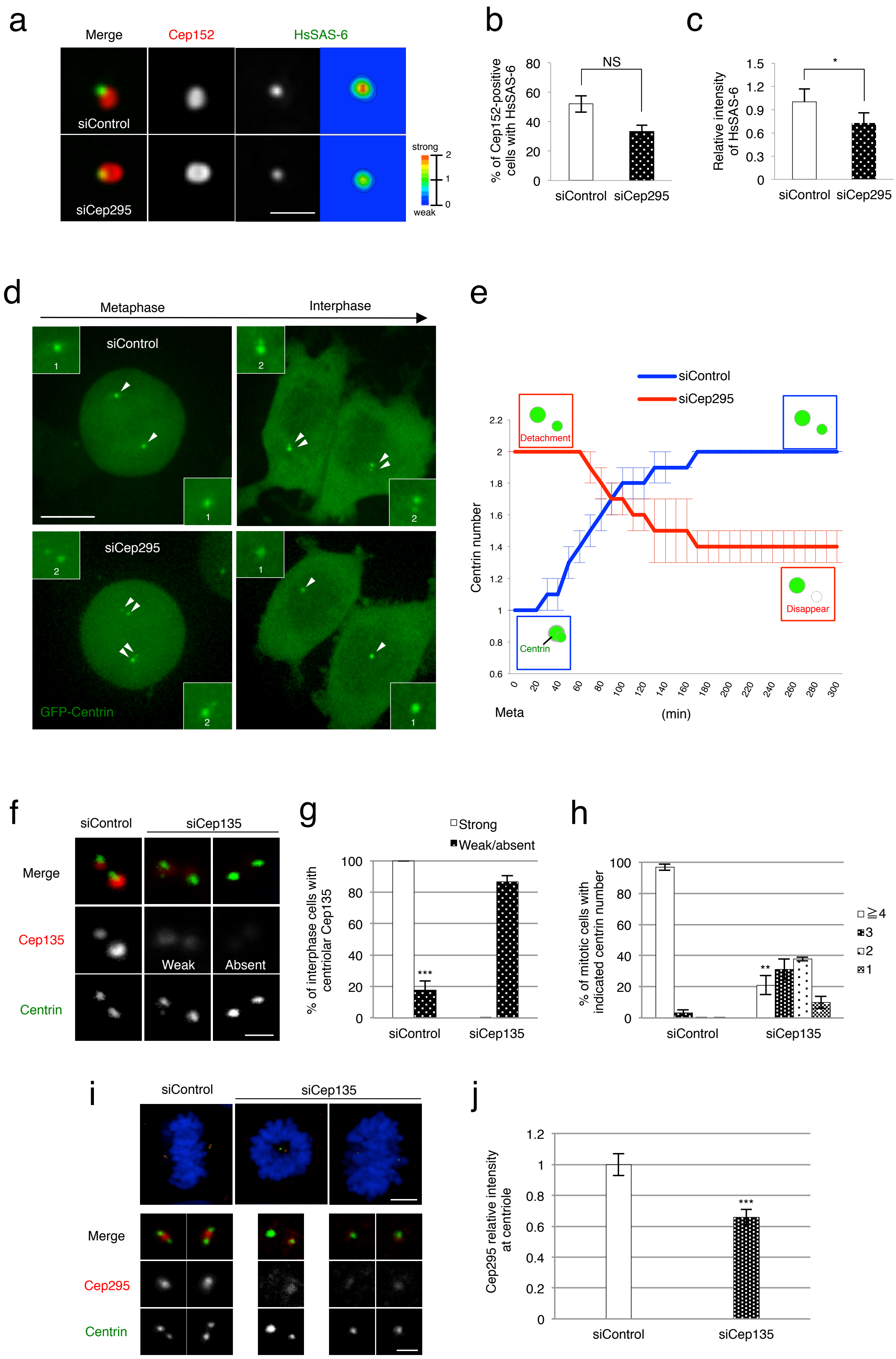

Supplementary Figure 4. Depletion of Cep295 leads to centriole disassembly after the defective centriole formation.

(a-c) Cep295 depletion results in defective recruitment of HsSAS-6. HeLa cells were transfected with control siRNA or Cep295 siRNA, and stained with antibodies against HsSAS-6 (green) and Cep152 (red). Cep152 was used as a mother centriole marker. Scale bar, 1 μm. (a) The right panels represent quantification of the local signal intensity of HsSAS-6. The local signal intensity was visualized in the indicated colors. (b,c) Histograms represent frequency of Cep152-positive cells with HsSAS-6 (in (b)) or relative signal intensity of HsSAS-6 (in (c)). Values are mean percentages ± s.e.m from three independent experiments (N > 15 for each condition). *, $P < 0.05$; NS, not significant (two-tailed *t*-test). **(d,e)** Depletion of Cep295 causes centriole disassembly. Live imaging of cycling HeLa cells expressing GFP-centrin1 (green) and treated with control siRNA or Cep295 siRNA. Representative images are shown in (d). Scale bar, 1 μm. Arrowheads point to GFP-centrin foci. Insets show magnified images of the fluorescent foci. The numbers in the insets indicate the number of GFP-centrin foci. (e) Quantification of the number of centrin foci in HeLa cells treated with control siRNA or Cep295 siRNA over time. Means ± s.e.m are shown (n =10). Time zero corresponds to the start of metaphase. **(f-h)** Cep135 depletion results in defective centriole formation. U2OS cells were transfected with control siRNA or Cep135 siRNA for 4 days. Scale bar, 1 μm. (f) Histograms represent frequency of interphase cells with the indicated intensity of centriolar Cep135 (g) or mitotic cells with the indicated number of centrin foci (h). Values are mean percentages ± s.e.m from three independent experiments (N = 30 for each condition). ***, $P < 0.001$; **, $P < 0.01$, (two-tailed *t*-test). **(i-j)** Cep135 depletion reduces Cep295 intensity at centrioles. U2OS cells were transfected with control siRNA or Cep135 siRNA for 4 days. Scale bar, 5 μm. Histograms represent the relative intensity of centriolar Cep295. Values are mean percentages ± s.e.m from three independent experiments (N = 20 for each condition). ***, $P < 0.001$, (two-tailed *t*-test).

Supplementary Figure 5

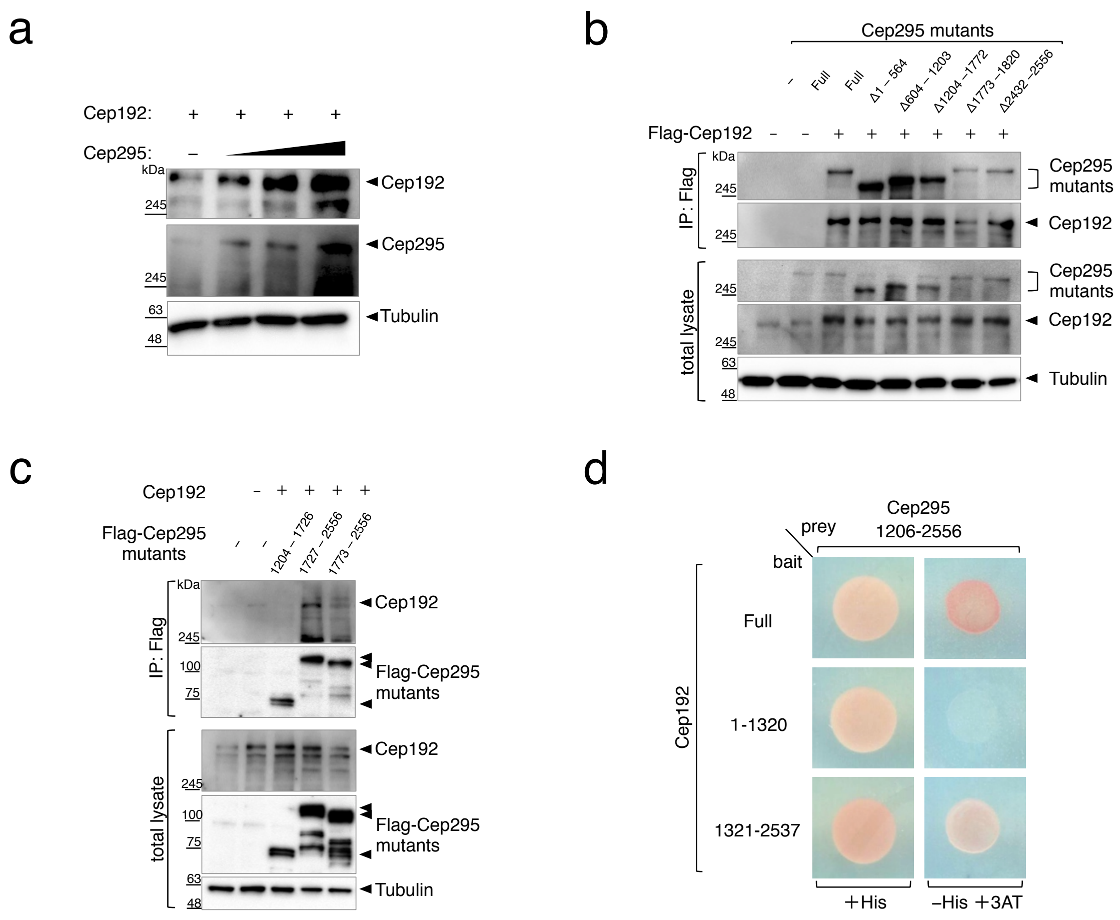

Supplementary Figure 5. Binding of Cep295 to Cep192 in human cells.

(a) Cep295 promotes Cep192 stabilization in a concentration-dependent manner. U2OS cells were transfected with the constant amount of Flag-Cep192 vector and also with various amounts of Flag-Cep295 vector. Total protein levels in each condition were analyzed by western blotting with antibodies against Cep192, Cep295 and tubulin (loading control). **(b,c)** Co-immunoprecipitation assay in U2OS cells testing interaction between Flag-Cep192 and the indicated Cep295 deletion mutants in (b), between non-tagged Cep192 and Flag-Cep295 deletion mutants in (c). The Flag-tagged proteins were immunoprecipitated using Flag beads from the cell lysate. Total cell lysates and IPs were analysed by western blotting using the indicated antibodies. **(d)** Yeast two-hybrid assay for testing interaction between the full-length or fragments of Cep192 and the C-terminal fragment of Cep295. The indicated clones were grown on the plates lacking histidine and containing 50 mM 3-AT.

Supplementary Figure 6

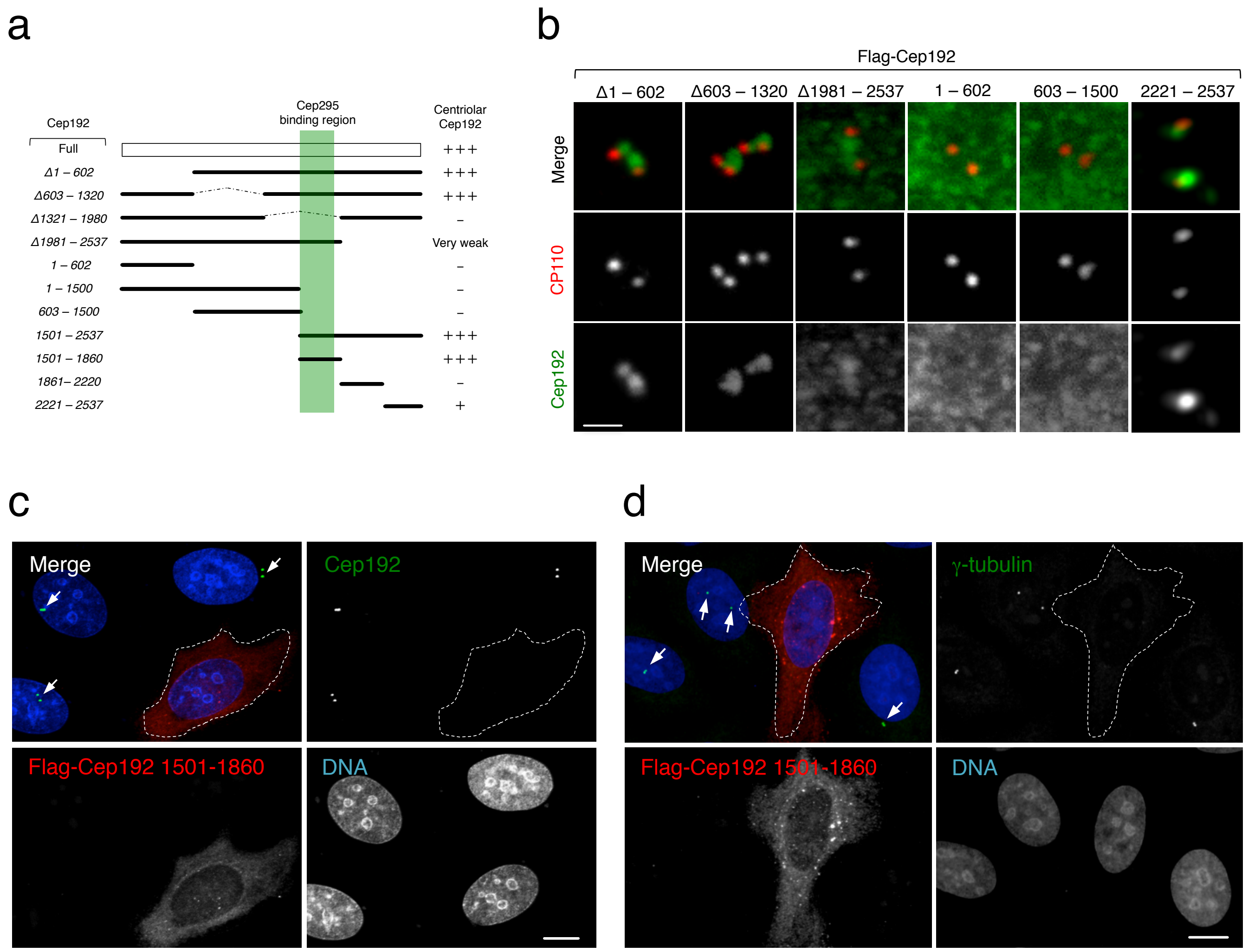

Supplementary Figure 6. Binding of Cep192 to Cep295 recruits Cep192 onto the daughter centriole.

(a) Schematic of full-length Cep192 and the deletion mutants used for immunofluorescence assay. The table shows centriolar localization of these constructs examined in U2OS cells. The minimal Cep295-binging region in Cep192 is represented in grey. **(b)** Centriolar localization of Flag-tagged Cep192 deletion mutants. U2OS cells expressing the indicated Flag-Cep192 mutant proteins were stained with antibodies against Flag (green) and CP110 (red). Scale bar, 1 μm. To precisely evaluate the function and localization of the Cep192 deletion mutants in human cells, it should be ideal to exogenously express those mutants in the absence of endogenous Cep192. However, there was a technical difficulty to perform RNAi-mediated reduction of endogenous Cep192 and overexpression of the Cep192 deletion mutants at the same time because this experimental condition caused severe defects in mitosis and toxicity for human culture cells. To avoid this, we decided to focus on the centriolar recruitment of Cep192 deletion mutants in the presence of endogenous Cep192. **(c,d)** Expression of the Cep192 fragment that binds to Cep295 inhibited centrosomal recruitment of γ-tubulin and endogenous Cep192. HeLa cells expressing the indicated Flag-Cep192 mutant proteins were stained with antibodies against Flag (red) and Cep192 or γ-Tubulin (green). Nuclei are shown in blue. Arrows point to Cep192 or γ-Tubulin foci. Scale bar, 10 μm.

Supplementary Figure 7

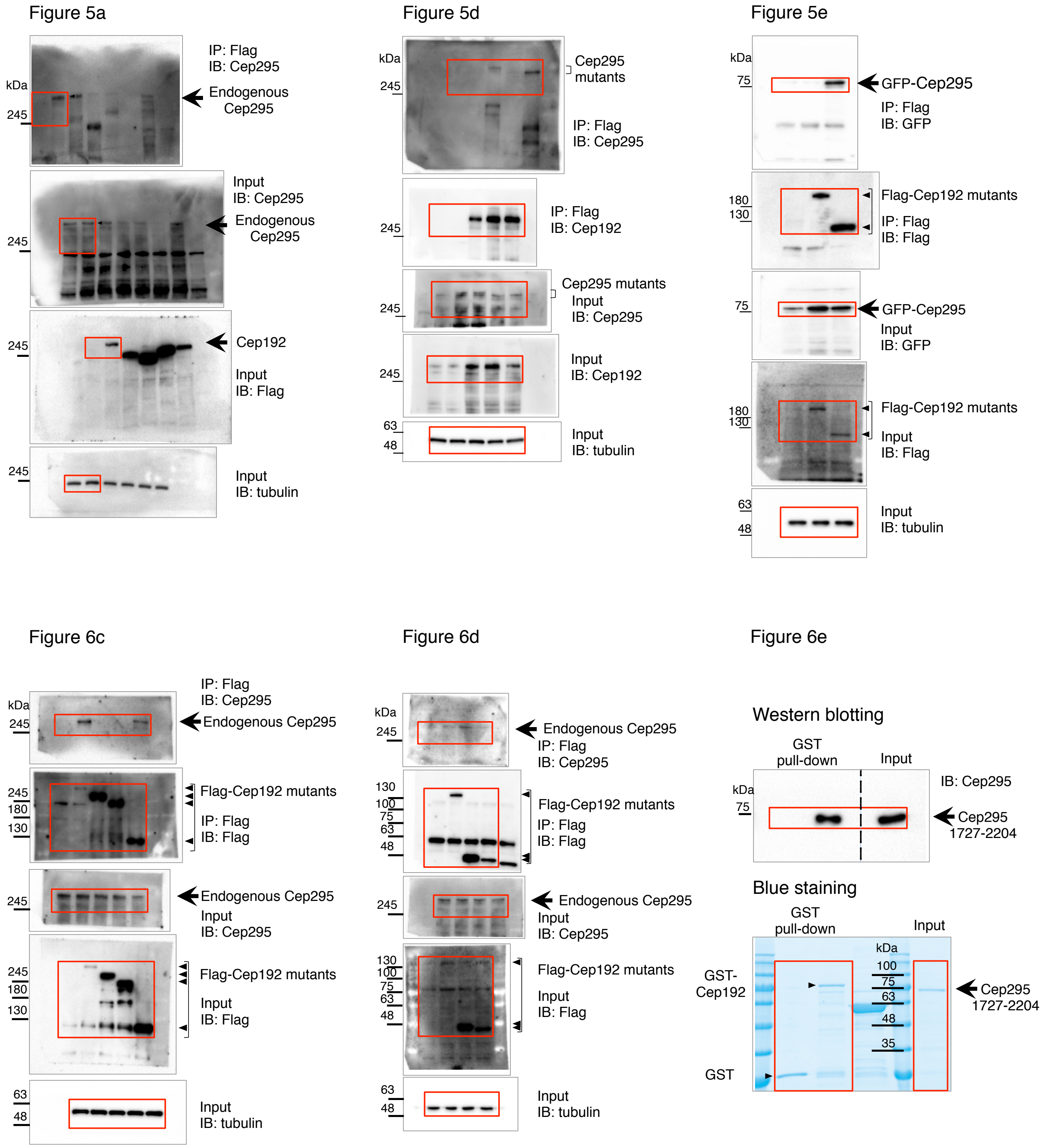

Supplementary Figure 7 continued

Figure S1c

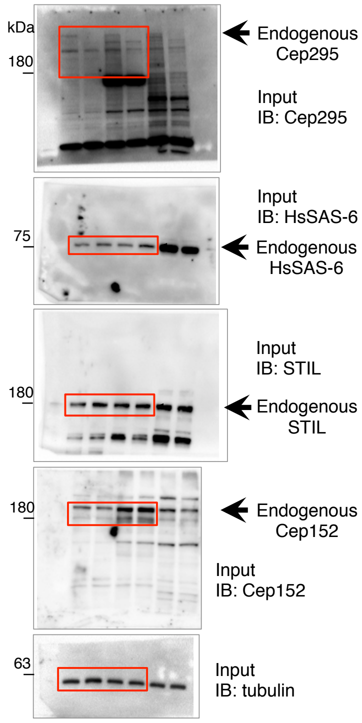

Figure S1e

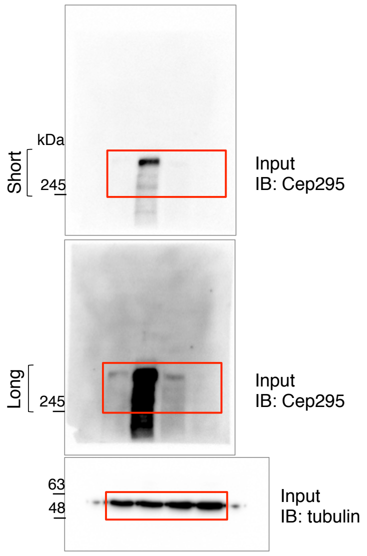

Figure S5a

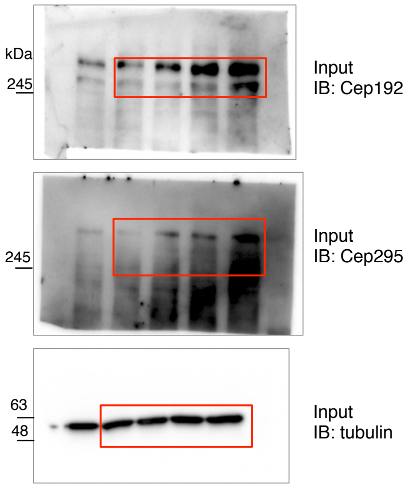

Figure S5b

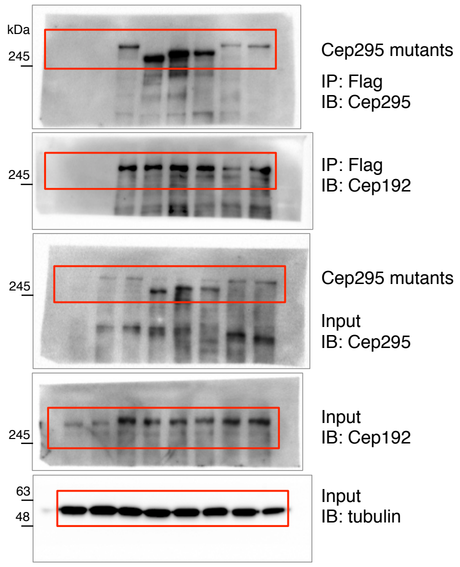

Figure S5c

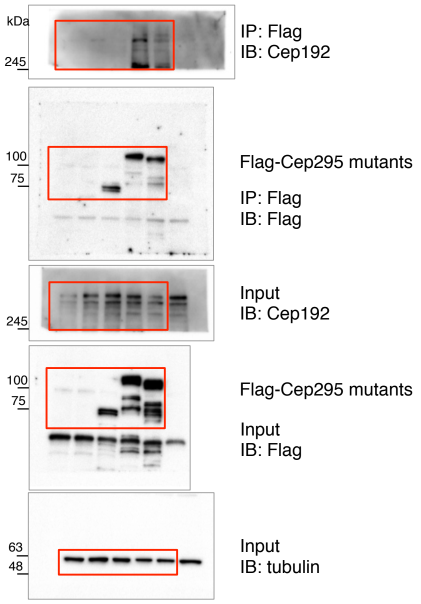

Supplementary Figure 7. Uncropped images of blots presented in the main paper.

Red boxes indicate the cropped regions. Molecular weight markers are indicated in kDa. IB; immunoblot, IP; immunoprecipitate
